# Gene Therapy in Orthopaedics: Progress and Challenges in Pre-Clinical Development and Translation

**DOI:** 10.3389/fbioe.2022.901317

**Published:** 2022-06-28

**Authors:** Rachael S. Watson-Levings, Glyn D. Palmer, Padraic P. Levings, E. Anthony Dacanay, Christopher H. Evans, Steven C. Ghivizzani

**Affiliations:** ^1^ Department of Orthopaedic Surgery and Sports Medicine, University of Florida College of Medicine, Gainesville, FL, United States; ^2^ Rehabilitation Medicine Research Center, Mayo Clinic, Rochester, MI, United States

**Keywords:** gene therapy, gene transfer, orthopaedics, viral vector, regenerative medicine, MSC mesenchymal stromal cell

## Abstract

In orthopaedics, gene-based treatment approaches are being investigated for an array of common -yet medically challenging- pathologic conditions of the skeletal connective tissues and structures (bone, cartilage, ligament, tendon, joints, intervertebral discs etc.). As the skeletal system protects the vital organs and provides weight-bearing structural support, the various tissues are principally composed of dense extracellular matrix (ECM), often with minimal cellularity and vasculature. Due to their functional roles, composition, and distribution throughout the body the skeletal tissues are prone to traumatic injury, and/or structural failure from chronic inflammation and matrix degradation. Due to a mixture of environment and endogenous factors repair processes are often slow and fail to restore the native quality of the ECM and its function. In other cases, large-scale lesions from severe trauma or tumor surgery, exceed the body’s healing and regenerative capacity. Although a wide range of exogenous gene products (proteins and RNAs) have the potential to enhance tissue repair/regeneration and inhibit degenerative disease their clinical use is hindered by the absence of practical methods for safe, effective delivery. Cumulatively, a large body of evidence demonstrates the capacity to transfer coding sequences for biologic agents to cells in the skeletal tissues to achieve prolonged delivery at functional levels to augment local repair or inhibit pathologic processes. With an eye toward clinical translation, we discuss the research progress in the primary injury and disease targets in orthopaedic gene therapy. Technical considerations important to the exploration and pre-clinical development are presented, with an emphasis on vector technologies and delivery strategies whose capacity to generate and sustain functional transgene expression *in vivo* is well-established.

## Introduction

In the early 1970’s, as the genetic bases for several debilitating inherited diseases were uncovered, gene therapy was viewed as a means to supplement, repair, or replace defective genes, whose products were either absent, functionally deficient, or pathogenic. Genetic modification of enough cells in the affected tissue(s) would mitigate the disease phenotype, and if stably inserted, lifelong benefit could be achieved. Exploratory work focused on life-threatening pediatric conditions whose etiology was linked to the absence or inactivation of one specific gene product.

Much like the hyperbole that currently envelopes all things “stem cell” (Caulfield et al., 2016; Sipp et al., 2018) in the 1990’s with the initiation of several clinical trials a similar wave of public attention and investigator allure accompanied the early stages of gene therapy (Verma, 1994; Friedmann, 2005). Crude marker studies describing the delivery of recombinant DNA to nearly every mammalian tissue incited a media deluge heralding imminent medical breakthroughs and life-changing cures. With time, it became increasingly clear that the development of effective gene therapies was far more difficult than turning a handful of cells blue or amending artificial disease in curated strains of rodents. In human trials, gene transfer was inefficient, producing too little protein for too short a time to be meaningful. Immune reactivity to the gene delivery vehicles (vectors) and therapeutic gene products, blocked or abbreviated transgene expression. Though the therapeutic potential remained, lack of clinical efficacy and in some cases serious treatment-related adverse events (Stolberg, 1999; Raper et al., 2003; Fehse and Roeder, 2008; Hacein-Bey-Abina et al., 2008), led to disillusionment and skepticism in both the scientific community and lay public (Evans, 2019).

Over the last 7–8 years or so, gene therapy has experienced a marked re-birth (Naldini, 2015) as several gene-based treatments have achieved clinical efficacy and received FDA approval (Dunbar et al., 2018). Much of this success can be attributed to in-depth studies (Collins and Thrasher, 2015; Naldini, 2015) of vector efficiency, biodistribution, safety and immunogenicity (Shirley et al., 2020). Importantly, these approved treatments span a wide range of conditions, including B-cell leukemia and lymphoma, melanoma, spinal muscular atrophy (Mendell et al., 2017), Leber congenital amaurosis (Russell et al., 2017), and lipoprotein lipase deficiency (Scott, 2015), and involve diverse gene delivery methods and technologies. Over the last year, vaccines against the SARS-CoV-2 virus, using mRNA and recombinant adenovirus (Altawalah, 2021) have been administered to hundreds of millions worldwide providing a compelling demonstration of the efficacy and safety of “gene-based” medications. With dozens of gene therapies in clinical testing, these successes have paved the way with both the FDA and the pharmaceutical industry for broad expansion of gene-based therapeutics over the next 10–15 years.

In the field of orthopaedics, gene transfer is being developed for targeted, sustained delivery of therapeutic gene products for treatment of common, yet problematic, multigenic pathologies of the skeletal connective tissues (bone, articular cartilage, tendon, ligament etc.) (Evans et al., 2021). As the role of these tissues is primarily structural, they’re predominately composed of collagen and extracellular matrix (ECM) components, often with low cellularity and limited vasculature. Healing is slow and often results in repair tissues of inferior composition and mechanical properties. Skeletal structures are also prone to chronic inflammatory and degenerative conditions that present significant clinical challenges.

**FIGURE 1:**
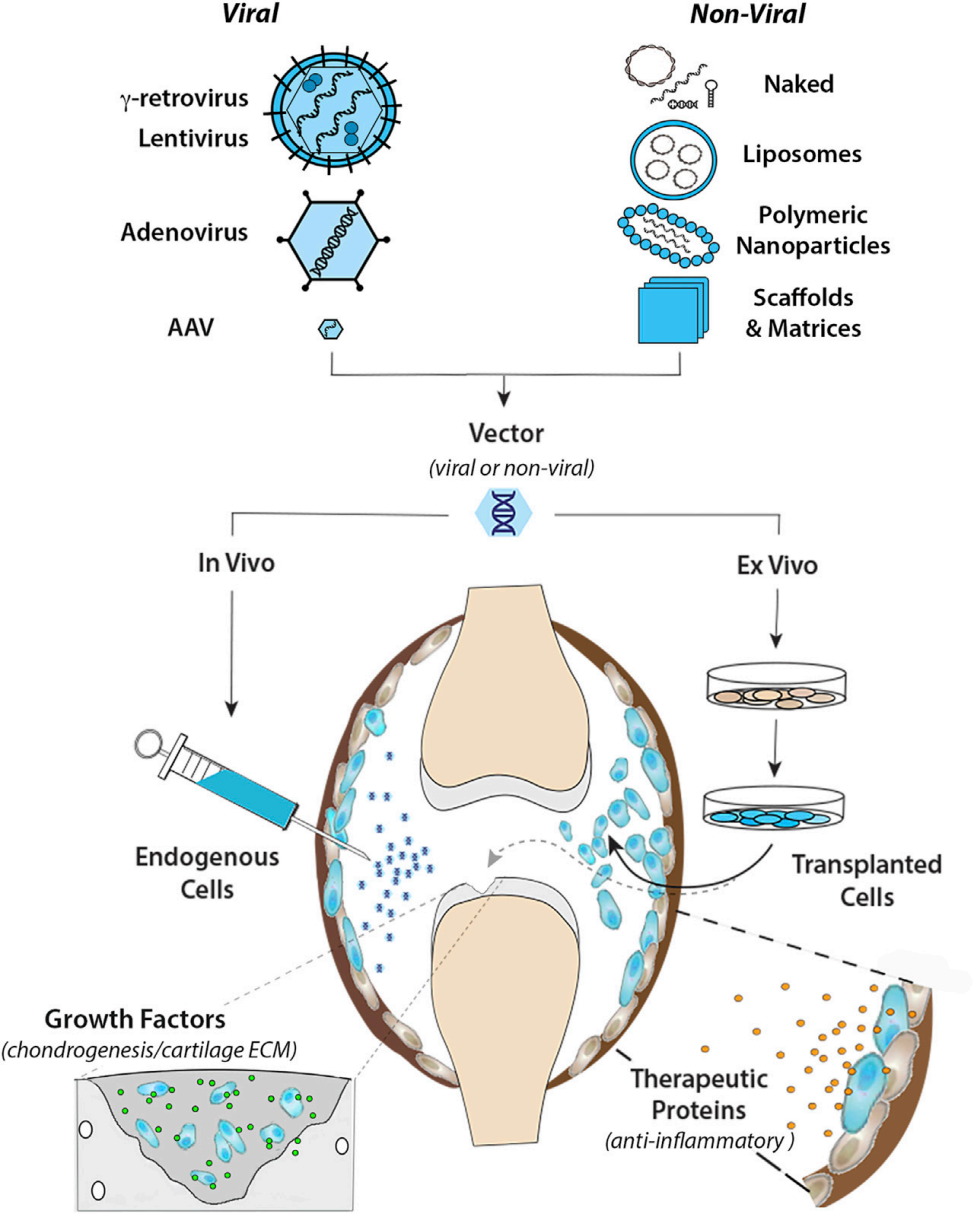
Intra-articular gene transfer- an archetypical model for orthopaedic gene therapy. (Top) Experimental strategies for delivery of therapeutic genes and nucleic acids to diseased or damaged tissues involve a variety of Viral and Non-Viral vector systems. The graphic on the left illustrates the basic structural and physical properties of the most widely used viral vector systems. The Retroviruses (γ-retrovirus and lentivirus) are relatively large (∼100 nm dia) enveloped viruses that “bud” from the surface of the infected cell. The outer envelope is composed of lipid bilayer acquired from the plasma membrane of the host cell during viral escape. Retroviral env (or VSV-G for pseudotyped virus) glycoprotein molecules transverse the outer envelope and are used for viral attachment to target cells. Two + strand copies of the RNA genome are encased in the protective nucleocapsid along with reverse transcriptase and integrase proteins that convert the RNA genome to DNA and integrate the provirus into the host genome. Adenovirus is a non-enveloped virus that replicates by lytic infection. The viral capsid is an icosahedron ∼90–100 nm in diameter that encases a linear dsDNA genome. Adenoviral fiber/knob complexes protrude from each of the 12 vertices of the icosahedron and are used for attachment to target cells. Following entry into the nucleus, the viral DNA remains episomal. Adeno-associated virus (AAV) is a comparatively simple, small non-enveloped, iscosahedral virus, ∼20 nm in diameter. The viral capsid houses a short (∼4.7 bp) ssDNA genome. Following infection, the genome is maintained episomally in concatemers formed by intermolecular recombination (Yang et al., 1999). Due to their inherent differences in biology and physical properties each viral vector is best suited to different types of applications and delivery strategies. To bypass the need for viruses for gene delivery, a wide range of non-viral systems (shown in the graphic on the right) are under investigation for their utility in orthopaedic applications. These non-viral systems utilize to varying extents, chemical modification of plasmid DNAs, soluble mRNAs, and RNAi molecules, which can be delivered “naked” in soluble form, or complexed with cationic lipids as liposomes or various polymers into nanoparticles to condense and protect the nucleic acids from degradation, prevent electrostatic repulsion and facilitate cellular uptake. Various carrier scaffolds and matrices are often employed to aid and prolong delivery to target cells. (Bottom) Once incorporated in an appropriate vector, the therapeutic gene or nucleic acid can be delivered to diseased or damaged tissues by either *in vivo* or *ex vivo* methods. For *in vivo* delivery, the vector is administered directly to tissues at the relevant site to modify the resident cell populations *in situ*. In the present example, the vector is injected intra-articularly into the synovial fluid of an arthritic joint to diffuse throughout the joint cavity and modify endogenous cells in the synovial lining (shown in blue) and/or articular cartilage. For *ex vivo* delivery, the vector is used to modify cells growing in culture, which can be administered locally to the site of disease or injury by different routes depending on the application. As indicated by the black arrow, the modified cells can be injected into the joint (or other relevant site) as a cellular suspension, to disperse and engraft in the local tissues to continuously express and secrete a therapeutic gene product (e.g., IL-1Ra, IL-10 etc.) into the local fluids and tissues to inhibit inflammatory signaling for an extended duration (right-hand inset). Alternatively, the modified cells can be incorporated into a support matrix and surgically implanted into a focal cartilage lesion (or other damaged tissue) (dashed gray arrow). Following delivery, the modified cells continually release specific growth factors to stimulate chondrogenic differentiation and cartilage matrix synthesis to facilitate repair by both the local and implanted cell populations (left-hand inset).

With advances in molecular technologies, numerous gene products (proteins and RNAs) have been identified with the potential to enhance tissue repair/regeneration and inhibit degenerative disease (Lyons and Rosen, 2019; Evans et al., 2021). However, the use of these molecules is often limited by the lack of methods for safe, effective delivery. With the exception of monoclonal antibodies, biologics typically have short half-lives *in vivo* (minutes to hours) (Evans et al., 2014). Skeletal tissue repair, though, is a prolonged process, often requiring weeks to months, while degenerative diseases, such as osteoarthritis (OA), are chronic, lifelong conditions. By delivering the coding sequences for these agents under independent control to cells in the pathologic environment, their biosynthetic machinery can be directed to overexpress the gene products for several weeks or months, and in some cases indefinitely (Figure 1) (Evans et al., 2021). The ability to target gene delivery specifically to sites of need, limits exposure of non-affected tissues to gene products with anabolic or immune suppressive activity. The success of proof-of-concept studies in rheumatoid arthritis (RA) (Bandara et al., 1993; Evans and Robbins, 1995b) and progression to clinical trial (Evans et al., 1996; Evans et al., 2005), inspired exploration of related strategies for multiple orthopaedic conditions, including skeletal fracture, OA, cartilage repair, intervertebral disk degeneration (IVDD), and tendon repair, among others (Evans and Robbins, 1995a).

Excepting the recent COVID-19 vaccines, current FDA-approved gene therapies target rare orphan diseases or involve methodologies tailored to individual patients. Due to the small numbers of recipients and the expense of vector production, genetic therapies presently come with a hefty price tag ranging from ∼$350,000 to >$2,000,000/patient (Dyer, 2020). The development of gene medicines for common, yet medically challenging disorders in orthopaedics and other specialties should reduce consumer costs dramatically and extend the benefits of gene-based therapeutics to the clinical mainstream (Evans et al., 2021).

## Assembly of a Gene Delivery Platform

Development of an effective gene-based therapy requires the integration of multiple biologic components into a treatment platform that addresses clinical need, without adverse consequences (Li and Samulski, 2020). Since the therapeutic agent is manufactured *in situ* by cells resident in the patient tissues, the pharmacokinetic profile (and, in turn, the efficacy of the approach) is dictated by the composition of the genetically-modified cell populations, their number, locations, metabolism and longevity (Watson Levings et al., 2018). When devising a gene-based therapy, *in vivo* tracking studies using cytologic marker genes (e.g., green fluorescent protein; GFP) are essential as they demonstrate the efficiency and distribution of gene transfer and expression in the tissue of interest (Li et al., 2021). These data are critical to the selection of appropriate vector systems and delivery methods and the types of gene products with the greatest therapeutic potential.

### Transgene Product- Secreted vs. Intracellular

Investigations of orthopaedic gene therapy have largely focused on the delivery of cDNAs encoding bioactive proteins that are secreted from the modified cells. Several advantages favor this approach. First, the coding regions of signaling molecules are often small and amenable to insertion in the limited space in most viral vectors. Second, a relatively small population of genetically modified cells can release transgene products into the surrounding fluids and tissues to affect regional cell populations in a paracrine manner. Further, the gene products released in conditioned media and biological fluids can be quantified by enzyme linked immunosorbent assay (ELISA), allowing compilation of pharmacokinetic profiles that define the functional parameters of the procedure (Watson Levings et al., 2018).

Alternatively, there are numerous gene products with therapeutic potential that function intracellularly. As there is no common mechanism by which exogenous proteins can be taken in by a cell and retain function, gene transfer is the only avenue by which certain types of molecules, e.g., nuclear receptors, kinases, transcription factors, RNAs etc. (Neefjes et al., 2020) can be exploited for clinical use. Since the direct effects of the gene product are delimited to the population of modified cells, the risk of adverse response from diffusion to non-target tissues is minimized. On the down-side, their overexpression intra-cellularly can often have negative consequences. For example, certain transcription factors essential for chondrogenic differentiation and bone formation (e.g., RUNX2 and SOX9), have been shown to induce deleterious skeletal phenotypes (Liu et al., 2001) and tumorigenic activation (Panda et al., 2021). From a technical standpoint, for an intracellular gene product to induce a meaningful response at the level of a tissue or organ, the bulk of the resident cell population must be genetically modified by the vector. Functional gene delivery of this magnitude is extremely challenging, even with highly efficient viral systems in small laboratory animals. Further, as efficacy is a function of the number, phenotypes, and locations of the modified cell populations (rather than total protein expression), exhaustive marker studies are necessary to assess and optimize the distribution of the vector and transduced cell populations in the target environment.

Mammalian cells are highly proficient at detecting and responding to extracellular stimuli. For most orthopaedic applications it’s far easier to direct the biology of the cells in a target tissue from the outside with exogenous cues and signaling molecules, than it is to reprogram sophisticated expression and signaling networks via ectopic expression of a transcription factor or interfering RNA (see below).

### Expression Cassette

**FIGURE 2:**
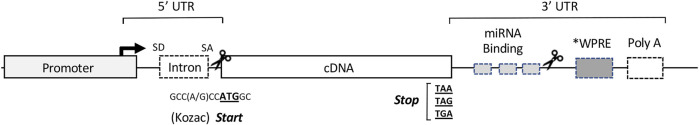
Organization of the cis-acting sequence elements in a typical expression cassette for therapeutic gene transfer. For most vector systems, both viral and non-viral, the expression cassette is designed to provide high-level synthesis of the transgene product independent of the regulatory constraints of the endogenous gene. The specific sequences of the elements in a particular cassette are often assembled from a variety of sources, both eukaryotic and viral (Keravala and Gasmi, 2021). The Promoter located at the 5′ end of the cassette, drives transcription of the therapeutic transgene (often the cDNA of a secreted protein). The transcription start site and direction of RNA synthesis are indicated by the arrow*.* The DNA sequences downstream from the promoter serve as the template for RNA synthesis, and the regions indicated represent the cis- acting RNA sequences in the 5′ and 3′ untranslated regions (UTRs) to enhance or regulate translation of the RNA transcript. The Intron: as a cDNA represents the protein coding sequence of a mature mRNA, an intronic sequence with flanking splice donor (SD) and acceptor (SA) sites is used to direct splicing of the primary transcript to enhance nuclear export and translation. The cDNA: the locations and template sequences of the translation start and stop codons are shown in bold. A consensus Kozac sequence flanks the ATG start codon, and during codon optimization is engineered into the sequence of the cDNA to enhance translation initiation and prevent cryptic starts at internal ATG (AUG) codons. miRNA Binding Sites: in the 3′ UTR, recognition sites for the binding regions of select miRNAs can be inserted to fine-tune or conditionally modulate mRNA translation in specific applications. WPRE: a woodchuck hepatitis virus post-transcriptional regulatory element or similar PRE can be inserted to enhance nuclear export and translation. Poly A: the polyadenylation signal at the 3′ end of the transcript serves as a cleavage site for the addition of the polyadenosine tract, which promotes nuclear export, mRNA stability and translation. Scissors: designate cloning sites for removal or insertion of cDNA(s) of interest. Regions internal to the cloning sites represent sequence elements specific to individual applications, while those outside are more generic and stably reside in the expression cassette of the vector.

Once a gene product is selected for study, commercial synthesis of the coding sequence permits codon optimization to enhance translation efficiency (Hanson and Coller, 2018) and additional sequence modifications to facilitate subcloning. Insertion of the designer transgene into an expression cassette with the appropriate cis-acting regulatory sequences enables efficient transcription, RNA processing and translation (Powell et al., 2015) in the milieu of the target tissue (Figure 2).

### Promoter

**FIGURE 3:**
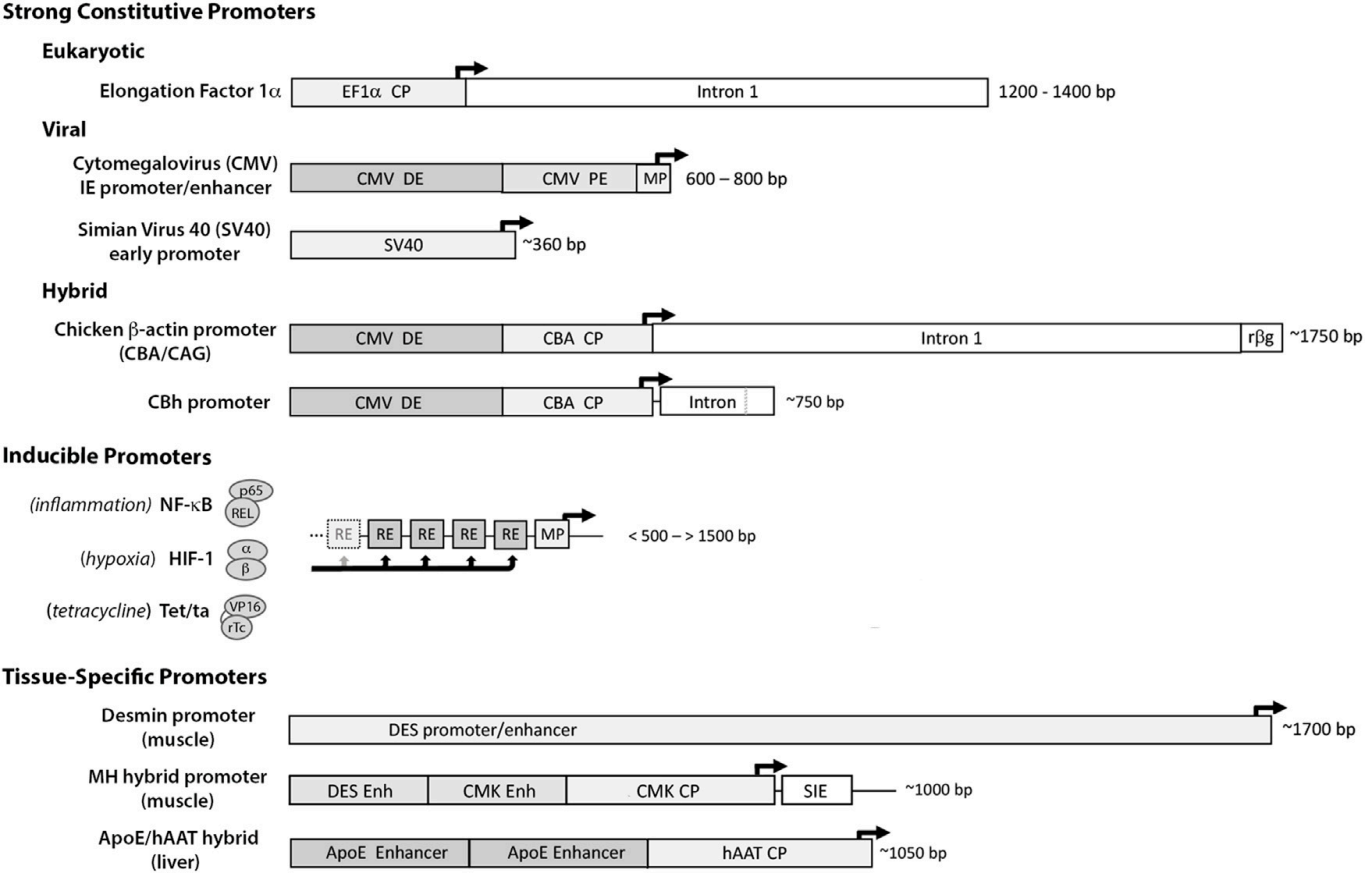
Promoter elements commonly used to drive therapeutic gene expression. Strong Constitutive Promoters: to compensate for limitations with gene delivery, most vector systems employ promoters with high basal activation for continuous high-level transgene expression. The diagram illustrates the differences in size and sequence components among some of the more widely used constitutive promoters. (The dark arrows indicate the transcription start site (TSS) and direction of RNA synthesis.) 1) the eukaryotic translation elongation factor 1α (EF1α) promoter: a short core promoter (CP) sequence lies upstream of the TSS with additional activation sequences located downstream in intron 1 of the EF1a gene (Wakabayashi-Ito and Nagata, 1994; Gopalkrishnan et al., 1999); 2) the cytomegalovirus (CMV) immediate early promoter/enhancer: composed of a minimal promoter (MP) that signals the TSS with short proximal and distal enhancer elements (PE and DE) immediately upstream (Prösch et al., 1996; Isomura et al., 2004) and 3) the early promoter from Simian Virus 40 (SV40): composed of a small minimal promoter with activation signals located in tandem 21 bp and 72 bp repeat sequences immediately upstream (Gendra et al., 2007). Other strong promoters common in the literature are Hybrids assembled from sequence elements of both eukaryotic and viral origin. Among these are 1) the chicken β-actin promoter (CBA/CAG) comprised of the CMV distal enhancer (DE) positioned upstream of the CBA core promoter, followed by the splice donor and enhancer elements from CBA intron 1 fused to the splice acceptor site of exon 3 of the rabbit b-globin gene (rβg) (Xu et al., 2001; You et al., 2010), and 2) the derivative CBh promoter composed of the CMV DE and CBA core promoter, with a hybrid intron immediately downstream composed of the splice donor site from CBA intron 1 fused to the splice acceptor site from minute virus of mice (MVM) VP intron (Gray et al., 2011). As each can provide high-level expression in the proper contexts, promoter selection is heavily influenced by size- of both the promoter and transgene and available space within vector. Inducible Promoters: for certain applications where conditional expression is desired, a wide range of synthetic inducible promoter systems are commercially available, or can be readily constructed/synthesized using the minimal CMV promoter as the transcription start site linked to an upstream array of up to 30 response/recognition elements (REs) (Ede et al., 2016) for potent transcription factor(s) induced by a particular change(s) in growth conditions, such as NF-kB (inflammation) or HIF1 (hypoxia) (Shibata et al., 2000). Alternatively transgene expression can be induced externally by the presence of a chemical agent, such as tetracycline which enables a Tet-binding transactivator (Tet/ta) to bind and interact with the TetO cis element (Loew et al., 2010). Tissue Specific Promoters: to prevent toxicity from transgene expression in off-target tissues, a number of tissue-specific promoters have been developed using the endogenous regulatory sequences. These elements are typically large, with weak transgene expression relative to ubiquitously active promoters, e.g., the muscle-specific desmin promoter/enhancer (DES) (Talbot et al., 2010). The muscle creatine kinase (CMK), Muscle Hybrid (MH) promoter designed *in silico* from muscle-specific transcription factor binding clusters is comprised of the desmin and CMK enhancer regions upstream of the CMK core promoter and followed downstream by a small intronic enhancer element (SIE) (Piekarowicz et al., 2019). *In vitro* and *in vivo* gene expression from the MH promoter was 2–4x that of CMV and >100x greater than the desmin promoter. A small liver-specific hybrid promoter comprised of core promoter for human α1 antitrypsin (hAAT) linked to upstream apolipoprotein E enhancer elements provides potent transgene expression in hepatocyte cultures and in liver, equivalent to CMV (Gehrke et al., 2003).

For most applications strong constitutively-active promoters are used to drive transcription of the transgene. These include the human translation elongation factor 1α (EF1α) promoter, the immediate-early cytomegalovirus (CMV) promoter/enhancer, the simian virus 40 (SV40) promoter and the chicken β-actin (CBA/CAG/CBh) hybrid promoters (Qin et al., 2010; Powell et al., 2015) (Figure 3). Each provides high basal activation for maximal constitutive gene expression. Though continuously active, expression can vary with cell type and metabolic state, and empirical testing *in vivo* is often necessary to identify the promoter(s) best-suited to the application.

Inducible promoter systems engineered with response elements for specific transcription factors can enable selective activation of transgene expression under defined conditions, such as inflammation or hypoxia, or the presence of an exogenous activator such as tetracycline (tet) as in the tet/on system (Qin et al., 2010; Powell et al., 2015) (Figure 3). Alternatively, tissue-specific promoters can limit transgene expression to a desired cell type or tissue, but low activity and/or large size can restrict their use. Given the challenges simply achieving functional levels of transgene expression *in vivo*, the formulation or use of inducible or self-regulating systems should only be considered when off-target effects or overproduction of the transgene product causes a deleterious response that cannot be addressed by reducing vector dose.

### Auxiliary Elements

Additional transcribed but non-coding sequence elements can be inserted into the template of the expression cassette (Figure 2), including synthetic introns for RNA splicing, polyadenylation sequences and post-transcriptional response elements (PRE) for enhanced nuclear export, translation and mRNA stability (Powell et al., 2015). Other regulatory elements (e.g., Kozac sequences, microRNA binding sites) can be engineered into the flanking untranslated regions (UTRs) of the transcript to fine tune mRNA translation for specific applications (Broderick and Zamore, 2011).

## Vectors for Gene Transfer

Since the uptake of exogenous nucleic acids by mammalian cells is extremely inefficient, a vector is required to ferry the expression cassette into target cells, facilitate nuclear trafficking and stabilize its activity. Vector development has followed two distinct tracks: 1) vectors derived from viruses, (Bulcha et al., 2021), and 2) those that are not (Zu and Gao, 2021). For the sake of clarity, viral-mediated gene transfer occurs through the process of transduction, while non-viral gene delivery via transfection.

### Viral Vectors

A virus is a biological entity comprised of genetic material (RNA or DNA) packaged in a protective shell (capsid). The relatively small viral genome codes for: 1) enzymes that preferentially express, replicate, and package its genetic material, and 2) the structural components of the viral particle. The remainder of the molecular components required for reproduction (e.g., polymerases, ribosomes, nucleotides, amino acids etc.) are provided by the host cell. Lacking the machinery for autonomous replication, procreation is contingent upon the ability of the virus to deliver its genetic material to permissive cell types with great proficiency. Natural selection over thousands of years, has allowed viruses to optimize their genome and capsid components for peak transduction efficiency. Thus, when devising a gene delivery strategy, appropriation of these naturally evolved systems is a logical approach. The challenges lie with engineering a recombinant form that’s technically manipulable, maintains efficient transduction and can be manufactured at high titer, while eliminating its ability to reproduce in the host and cause pathology (Bulcha et al., 2021).

In general, a viral vector is created by removing the coding sequences from its genome, while leaving in place the non-coding elements required for replication and packaging into the viral capsid. Since the genome length of the wild type (wt) virus approximates the maximum amount of genetic material that can be packaged in the viral capsid, removal of viral genes creates room for an exogenous expression cassette. Transfer and expression of the viral coding sequences in a complementing cell line allows for selective replication and packaging of the vector DNA. The resulting virions can infect and transduce target cells but can only replicate in the complementing cell line. Removal of viral coding sequences from the vector genome, precludes their expression in transduced cell populations, reducing immune recognition and elimination. As each viral vector has a different tropism and transduction pathway, the vector and expression cassette must be tailored to the therapeutic needs of the target disease. The following sections describe the salient features of the viral vectors most common in clinical gene therapy: adenovirus (Ad), adeno-associated virus (AAV), γ−retrovirus and lentivirus.

### Adenovirus

Vectors derived from adenovirus (Ad) have been widely used in orthopaedic research due to their broad host range, high-level of infectivity, and ease of propagation. wtAd is common in nature and generally associated with self-limiting respiratory infections but can also infect the brain and bladder. The viral capsid is a non-enveloped icosahedron ∼80–100 nm in diameter that encases a linear, double stranded (ds)DNA genome ∼35 kb in length. Figure 1 The Ad genome is flanked on either end by inverted terminal repeat (ITR) sequences required for replication; a short *psi* (Ψ) sequence marks the DNA for packaging into the viral capsid. The wt genome encodes ∼35 viral proteins, sequentially expressed in the early (E) and late phases of viral infection and replication (Bulcha et al., 2021).

Early generation vectors were created by removing the immediate early E1A and E1B genes, and later the E3 gene, creating room for an expression cassette of up to 7.5 kb. Since E1A is required for transcription of the other viral genes, removal of the E1 locus renders the vector replication-deficient in normal cells. The vector is propagated in 293 cells (Graham et al., 1977), which stably harbor the left-hand end of the wt adenoviral genome, and constitutively express the E1A and E1B proteins (Louis et al., 1997). Infection of 293 cells with recombinant Ad provides the E1 proteins in trans, allowing its replication.

Ad vectors provide several functional and technical advantages which have made them the workhorse system in studies of orthopaedic gene therapy: 1) broad tropism, 2) efficient transduction of dividing and non-dividing cells, 3) non-integrating genome, 4) high-level transgene expression with rapid onset; 5) relatively large packaging capacity, and 6) straightforward methods of propagation. Distinct limitations are their propensity to provoke inflammation and immune recognition of transduced cells *in vivo*. Despite removal of the E1 locus, leaky readthrough transcription allows low-level expression of the residual viral coding sequences and the activation of antigen-specific CD8^+^ cytotoxic T lymphocytes (CTL) that selectively kill the transduced cell populations (Yang et al., 1994). Within 3–4 weeks of vector delivery, initially robust transgene expression is progressively extinguished as the Ad-infected cells are found and eliminated (Shirley et al., 2020). In certain disease/injury models, a few weeks of high-level expression is sufficient to provide a robust demonstration of the bioactivity and therapeutic potential of a candidate gene product *in vivo*.

To reduce the immunogenicity of Ad-infected cells, “gutted” or “high-capacity” (HC) vectors have been developed in which all viral coding sequences have been removed from the genome, leaving only the flanking ITRs and the *psi* packaging sequence. HC vectors can accommodate up to 36 kb of exogenous DNA but require co-infection with a helper adenovirus for replication. While removal of the viral coding sequences reduces the immunogenicity of transduced cells and extends transgene expression *in vivo*, innate immune responses to the capsid protein and adenoviral infection can still cause the vector to be inflammatory and in certain contexts will lead to its elimination (Muruve, 2004; Carlin, 2019). Removal of the helper virus from vector preparations presents additional challenges (Alba et al., 2005). Further, due to the prevalence of wt adenovirus in nature, much of the human population is seropositive for neutralizing antibodies (NAbs) against one or more human variants, limiting the use of the most common Ad vectors in humans (Mast et al., 2010). To circumvent immune inactivation, vector systems have been developed from non-human variants common in other species. Notably Ad vectors developed from variants found in chimpanzees are used to deliver and express the coding sequence for the SARS-CoV-2 spike protein and are in widespread use as vaccines against COVID-19 (Barrett et al., 2021; Ewer et al., 2021).

### Adeno-Associated Virus

Recombinant (r)AAV is another non-integrating viral vector capable of transducing both dividing and non-dividing cells (Li and Samulski, 2020). Compared to other prominent viral systems, rAAV is accepted as the least toxic, and induces relatively low innate and adaptive immune responses against transduced cells (Zaiss et al., 2002; Somanathan et al., 2010). Though its genome remains episomal, rAAV can support long-term (>10 years) transgene expression in quiescent cells *in vivo* (Lebherz et al., 2005; Rivera et al., 2005; Nathwani et al., 2014). With a favorable safety profile in over 200 clinical studies, rAAV is the preferred vector system for human protocols involving *in vivo* gene delivery.

wtAAV is a small (∼20 nm diameter), non-enveloped, single-stranded (ss)DNA virus, that is non-pathogenic in humans. Naturally replication defective, wtAAV requires co-infection with a second virus (e.g., adenovirus or herpes simplex virus) to provide helper functions necessary for replication (Xiao et al., 1998; Linden and Berns, 2000). The 4.7 kb genome harbors four open reading frames (ORFs) including the *rep* and *cap* genes necessary for its replication and packaging. Short inverted terminal repeat (ITR) sequences, 145 nt in length, flank the viral genome and internally base-pair to form hairpin structures necessary for priming genomic replication and packaging (Linden and Berns, 2000). As the ITRs are the only required sequence elements for vector replication and packaging, the vector genome is remarkably simple, and comprised only of an expression cassette with an ITR on either end. The packaging limit of ∼5 kb precludes the use of large cDNAs and promoter sequences. The viral vector is propagated by co-transfection of 293 cells with the vector plasmid and plasmids harboring the *rep* and *cap* genes and the adenoviral helper functions.

At least 12 natural serotypes and more than 1,000 naturally-occurring wtAAV variants have been identified, which preferentially target various cell surface glycans and secondary receptors (Mietzsch et al., 2014). AAV serotype 2 (AAV2), the most prevalent variant in humans, uses heparan sulfate proteoglycan as a primary binding receptor (Summerford and Samulski, 1998), while AAV5 binds N-linked sialic acid and AAV9 targets galactose (Li and Samulski, 2020). Once bound to the cell surface, secondary receptors mediate viral entry, where the virus is trafficked through late endosomal and lysosomal compartments before being shuttled into the nucleus and unencapsidated. The ssDNA genome of conventional AAV vectors, requires synthesis of the complementary DNA strand before it can be recognized by the transcriptional apparatus of the nucleus (Li and Samulski, 2020). The ITRs facilitate inter- and intra-molecular recombination to form concatenated dsDNA circles, which enable the vector genomes to be maintained as episomal elements (Dudek et al., 2018). In adult mesenchymal tissues, where the resident cell populations are largely quiescent, the requirement for second strand DNA synthesis can be prohibitive to transgene expression. However, deletion of the terminal resolution sequence (*trs*) from one ITR provides for the synthesis of genomes with covalently linked + and – DNA strands. Intra-molecular base-pairing forms double-stranded self-complementary (sc)AAV genomes that are fully functional at the time of infection (McCarty et al., 2001; McCarty et al., 2003; Li and Samulski, 2020) with substantially higher transduction efficiency in mesenchymal cells and rapid onset of expression (Kay et al., 2009). Since the sc modification doubles the size of the vector genome, the packaging limit is cut in half to ∼2.5 kb (McCarty et al., 2001; McCarty et al., 2003) which further restricts the size and composition of the expression cassette.

Though non-pathogenic, childhood infections from wtAAV are common and often induce life-long production of capsid-specific NAb. Depending on location, anywhere from 30%–70% of the human population have circulating NAb to one or multiple AAV variants (Calcedo et al., 2009). The ability to cross-package (or pseudotype) the AAV vector genome in different capsids alters its tropism and provides the opportunity to increase gene transfer efficiency in specific tissues. Cross-packaging also provides the potential to evade pre-existing capsid-specific NAb from natural infection or treatment with rAAV (Li and Samulski, 2020). The generation of designer AAV capsids with enhanced properties for specific applications using rational design (Li et al., 2012; Wang et al., 2019), directed evolution, (Marsic et al., 2014), and in silico approaches (Zinn et al., 2015; Smith et al., 2016; Li and Samulski, 2020) is currently an area of intense investigation.

### Retroviral Vectors

Vectors derived the retrovirus family (e.g., γ-retrovirus, and lentivirus) are spherical enveloped viruses ∼100 nm in diameter with genomes composed of two copies of sense (+) strand RNA (Robbins et al., 1994; Vogt, 1997). Figure 1 In contrast to non-enveloped viruses (e.g., adenovirus and AAV), which reproduce by lytic infection and release thousands of viral progeny in a burst that kills the host cell, retroviruses are enveloped and reproduce by continuous budding from the surface of the infected cell. Though the genome is encased in a protective capsid, the outer envelope is comprised of lipid bilayer appropriated from the plasma membrane of the host cell.

γ-retroviruses, which harbor only three genes are considered to be simple, while lentiviruses with nine overlapping coding regions are classified as complex (Bulcha et al., 2021). The retroviral genome is flanked by sophisticated long terminal repeat (LTR) sequences containing strong promoter/enhancer elements that drive transcription of the entire viral genome. Other cis- acting elements include sequences for replication priming and a *psi-* sequence for encapsidation (Beasley and Hu, 2002). All retroviral genomes contain three core genes: *gag, pol,* and *env* (Hanawa et al., 2002). The group associated antigen (*gag*) gene codes for the protective capsid. The *env* gene codes for glycoproteins that transverse the viral envelope and bind to surface receptors of target cells (Maetzig et al., 2011). For both retroviral vectors, the endogenous *env* gene is often replaced with the coding sequence of the vesicular stomatitis virus glycoprotein (VSV-G) (Hanawa et al., 2002), which dramatically expands the tropism, enhances infectivity, and increases the stability of the vector particle. The *pol* gene codes for reverse transcriptase (RT) and integrase proteins that are carried within the viral envelope (Hanawa et al., 2002). Viral RT converts the RNA genome to dsDNA, which the integrase inserts into the cellular genome as a provirus, preferentially targeting regions that are transcriptionally active (Mitchell et al., 2004). Retroviral vectors are highly infectious, elicit relatively weak immune responses, and vector integration provides the potential for stable expression of the transgene, which can be amplified with subsequent cell divisions *in vitro* and *in vivo* (Naldini, 2011).

### γ-Retrovirus

The vector derived from Moloney murine leukemia virus (MoMLV) was among the first developed from γ-retrovirus, and the first used successfully in a clinical trial (Mann et al., 1983; Maetzig et al., 2011). Unable to penetrate the nuclear envelope of an infected cell, γ-retroviruses can only access the host genome during mitosis, when the nuclear envelope is disassembled (Mann et al., 1983; Miller et al., 1990). This limits their host range to cells that are actively dividing and confines their use to *ex vivo* applications. In early vectors, the *gag, pol*, and *env* genes were replaced by the cDNA of interest and expression was driven by the promoter/enhancer of the 5′ LTR (Maetzig et al., 2011). The vector was propagated in complementing cell lines modified to stably express the *gag, pol*, and *env* genes. As one of the earliest viral vector systems, γ-retroviral vectors have been used in numerous clinical trials, including gene therapy for RA (Evans et al., 1996).

γ-retroviral vectors preferentially integrate near the transcription start sites of active genes, with a particular affinity for proto-oncogenes (Wu et al., 2003), which brings the potential for insertional mutagenesis and oncogenic activation of the infected cell (Mitchell et al., 2004). Insertion of the promoter/enhancer elements of the vector LTRs near the start site of a cell cycle gene can hyper-induce its expression and lead to clonal proliferation. In one of the earliest gene therapy trials, a γ-retroviral vector was used to modify hematopoietic stem cells for correction of X-linked SCID. Though the treatment stably reversed disease in 9 of 10 male infants, four of the boys went on to develop T-cell acute lymphocytic leukemia. Analysis of the leukemic cells revealed vector integration adjacent to proto-oncogenes LMO2 and BMI (Hacein-Bey-Abina et al., 2008; Howe et al., 2008; Thornhill et al., 2008). Based on similar events in other trials, self-inactivating (SIN) vectors were created in which the LTR enhancers are deleted/inactivated reducing the risk of vector-induced oncogenesis and the generation of replication competent retrovirus (RCR) (Yu et al., 1986; Suerth et al., 2010).

### Lentivirus

Following infection, lentiviruses employ active transport mechanisms to traverse the pores of the nuclear envelope and access the host chromosomes. This allows recombinant lentiviral vectors to transduce both dividing and non-dividing cells with similarly high efficiency (Roe et al., 1993; Lewis and Emerman, 1994) and extends their use to *in vivo* applications. Although lentiviruses preferentially integrate in transcriptionally active chromatin, they typically target gene bodies over start sites, which reduces the risk of genotoxicity (Schröder et al., 2002; Mitchell et al., 2004). Risk can be further decreased by targeting quiescent cells with inactive cell cycle genes (Durand and Cimarelli, 2011). Due to increased versatility and safety, recombinant lentivirus has emerged as the preferred retroviral system for clinical gene delivery, *ex vivo* applications in particular.

The first and most often used lentiviral vector was derived from human immunodeficiency virus I (HIV1) (Naldini et al., 1996; Naldini, 2011), the agent responsible for acquired immune deficiency syndrome (AIDS), and tremendous effort was invested to minimize the potential for adverse effects (Naldini et al., 1996; Dull et al., 1998). In addition to the core retroviral genes, the HIV1 genome contains four virulence factor genes (*vif, vpr, vpu*, and *nef*) and two regulatory genes (*rev* and *tat)* with overlapping coding sequences. In the current third-generation vectors, all but *rev*, which is essential for vector replication, have been deleted (Dull et al., 1998). Since the tropism of HIV1 is restricted to CD4^+^ T helper cells, the vector is commonly pseudotyped with VSV-G. To reduce the possibility of generating RCR by intermolecular recombination, the viral elements required for vector production are delivered to packaging cells in four separate plasmids: 1) a transfer vector, 2) a packaging plasmid harboring the *gag* and *pol* genes from HIV1, and separate expression plasmids containing the genes for 3) *rev* and 4) VSV-G (Hanawa et al., 2002). The expression cassette of the transfer vector can accommodate genetic payloads of 8–9 kb, and is flanked by LTRs with inactivated enhancers to create a SIN packaging system (Naldini, 2015). Transgene expression is driven by an exogenous promoter (often CMV) rather than the native LTR. Insertion of the woodchuck post-transcriptional regulatory element (WPRE) is used to enhance RNA stability and translation.

## Non-Viral Gene Delivery

Non-viral gene transfer initially focused solely on the delivery of plasmid DNAs (Zu and Gao, 2021); the field has since grown to include an assortment of nucleic acids composed of both RNA and DNA.

### Plasmid DNA

Historically, the pursuit of non-viral gene transfer has been motivated by its perceived advantages over viral-based systems (Hardee et al., 2017), that included: 1) increased safety, 2) ease of manipulation, 3) reduced production costs, 4) lack of immune response, and 5) large payload capacity. With advances in viral technologies, the extent to which these advantages still exist is highly questionable. While exogenous nucleic acids do not provoke adaptive immune responses, they are potent inducers of innate immune pathways (Turvey and Broide, 2010) and are characteristically inflammatory following delivery *in vivo*. Regarding safety, available data from >200 clinical trials indicate that AAV-based vectors administered at moderate doses are safe, well-tolerated and efficacious (Kuzmin et al., 2021). Although large plasmids containing multiple expression cassettes can be assembled fairly readily, transfection efficiency is inversely proportional to the size of the construct, such that plasmid uptake and expression drops precipitously with constructs >3 kb in length and is most efficient with minicircles of 650 bp or less (Kreiss et al., 1999; Yin et al., 2005). Considering the functional limitations of non-viral gene transfer *in vivo* (i.e., low transgene expression of brief duration), lower production cost is a non-issue.

DNA (and RNA) has a pronounced negative charge that inhibits diffusion through the plasma membrane of target cells. Complexation with cationic agents is used to mask the electrostatic charge and condense the nucleic acid and facilitate uptake (Meng et al., 2017). The complexes must enter the cell through endocytosis or pinocytosis and passively find their way to the nucleus, then traverse the nuclear envelope before they can be transcribed (Zu and Gao, 2021). Much like γ-retrovirus, transfection efficiency is far higher in mitotic cells, which strongly favors *in vitro* applications. Technologies such as electroporation, hydrodynamic injection, and ultrasound can enhance uptake *in vivo*, but are difficult to administer in larger animals/tissues and can provoke significant damage. Regardless of the method of delivery, non-viral transgene expression *in vivo* is extremely modest and transient, which limits its useful applications to vaccinations and *ex vivo* procedures.

### mRNA

The development of methods for delivery of soluble mRNA, and recently its widespread application in SARS-CoV-2 vaccines, have moved this “gene-based” strategy to the forefront of the non-viral field. Soluble mRNA has a markedly higher transfection efficiency than plasmid DNA. Once internalized by the cell mRNA is immediately available for translation, bypassing the need for nuclear trafficking, translocation, transcription, RNA processing and nuclear export (Meng et al., 2017; Balmayor and Evans, 2019).

As with plasmid DNA, soluble mRNAs are complexed with cationic lipids or polymers to enhance stability and transfection. As a single-stranded polymer, RNA can internally base-pair to form double stranded (ds) regions, whose recognition by intra-cellular toll-like receptors (TLRs) triggers innate immune activation and release of inflammatory cytokines (Meng et al., 2017). The addition of a 5′ cap and poly-A tail, and various chemical modifications improve mRNA stability and translation, and reduce inflammatory activation (Balmayor and Evans, 2019). Though soluble mRNAs have a limited life-span intra-cellularly, they can persist for several days, providing a burst of local protein expression in the context of a moderate inflammatory response. This pattern is useful for vaccination and applications where repeat dosing is straightforward and inflammatory signaling is a component of early-stage healing or repair (Balmayor and Evans, 2019).

### RNA Interference

RNA Interference (RNAi) is a broad term encompassing gene regulation by small, non-coding RNAs that selectively base-pair with target mRNAs to inhibit translation (Lam et al., 2015). In each case a short guide sequence on the RNAi molecule directs an assembly of ribonucleoproteins, the RNA-induced silencing complex (RISC) (Ichim et al., 2004), to complementary sequences on the mRNA target. Depending on nature of the RNA duplex, the mRNA will either be cleaved (Elbashir et al., 2001), degraded, or its translation repressed.

Micro-RNAs (miRNAs) are endogenously produced to modulate gene expression in a cell type-specific manner. With guide sequences of ∼21 nt, miRNAs bind imperfectly to sequences in the 3′ untranslated region (UTR) of mRNAs to inhibit translation or destabilize the RNA leading to its degradation. Incomplete or partial base-pairing allows one miRNA to inhibit the translation of dozens of mRNAs. Small-interfering RNAs (siRNAs) and short-hairpin RNAs (shRNAs) use their guide sequences (∼19–22 nts) to base-pair with 100% identity to the coding regions of mRNAs, inducing cleavage and degradation.

Therapeutic use of synthetic RNAi molecules is based on the targeted silencing of disease-causing gene products. While potentially useful against infections, targeted gene inhibition is not well-suited to the activation of repair pathways or treatment of heterogeneous multigenic diseases, such as OA. As with other intra-cellular approaches, efficacy requires that a large majority of the cells in the diseased tissues acquire and maintain the RNAi molecules at functional levels. Soluble RNAs have limited intracellular half-lives and only persist for few days. For more sustained activity, their sequences can be incorporated into a viral vector and synthesized continuously in modified cells as shRNAs or miRNA mimics (Cullen, 2005; Silva et al., 2005; Zeng et al., 2005), but efficacy is still contingent upon highly efficient transduction of the cells in the target tissue. Since gene inhibition is based on sequence complementarity, limited incidental base-pairing with the UTRs of an unintended mRNA(s) can suppress its translation and lead to undesirable off-target effects, including death in laboratory animals (Jackson et al., 2003; Lin et al., 2005; Jackson et al., 2006; Rao et al., 2009; Gagnon and Corey, 2019). Off-target suppression is increased by RNAi molecules at supraphysiologic levels (Rao et al., 2009; Gagnon and Corey, 2019). Since the use of RNAi in vivo requires high vector doses, overexpression of RNAi gene products in a sub-population of cells is an inevitable consequence, and brings the risk of patient morbidity from prolonged off-target gene silencing (Grimm et al., 2006).

As stated earlier, RNAi is exceedingly difficult to control on a per-cell basis and is incompatible with regenerative strategies. Moreover, chronic degenerative conditions, such as OA and IVDD have complex multifactorial etiologies involving multiple diverse signaling mechanisms and redundant activation pathways. In the face of such complexity and heterogeneity of disease, it’s extremely unlikely that the downregulation of a single gene product in a minority subpopulation of cells in a pathologic environment can meaningfully impact disease progression.

## Gene Editing- CRISPR/Cas9

Over the last decade, technologies for targeted editing of eukaryotic genomes have been developed from bacterial defense systems that utilize Clustered Regularly Interspaced Short Palindromic Repeat (CRISPR) sequences (Barrangou et al., 2007). The spacer sequences between the repeats are transcribed and incorporated into protein complexes to create RNA-guided nucleases that selectively cleave and inactivate the DNAs of invading viruses and plasmids (Jinek et al., 2012). The CRISPR system used for genomic editing is comprised of two core components: 1) a small guide RNA (gRNA) ∼125 nts in length, and 2) a CRISPR-associated nuclease (Cas9), which combine to form a ribonucleoprotein (RNP) complex (Mali et al., 2013). Once bound by Cas9, an 18–20 nt sequence at the upstream 5′ end of the gRNA directs the RNP complex to the complementary sequence(s) in the host genome with high specificity. RNP binding and DNA cleavage at the target locus in the genome requires 1) sequence complementarity with the gRNA targeting domain and 2) the presence of a short 3 base-pair sequence (e.g., 5′ NGG) on the opposite DNA strand immediately downstream from the target site (termed the “Protospacer Adjacent Motif,” or PAM). Once bound to the target locus, the Cas9 nuclease makes a double strand break (DSB) in the genomic DNA 3–4 bp upstream of the PAM. The fractured chromosome triggers the induction of endogenous DNA repair pathways [i.e., Non-Homologous End Joining (NHEJ) or Homology Directed Repair (HDR)] whose respective activities are co-opted for specific editing functions.

### Targeted Gene Knockout

As the principal repair pathway for DSBs, NHEJ is an efficient, but error-prone process that often generates small (1–10 bp) heterogeneous nucleotide insertions or deletions (“indels”) at the repair site as the DNA ends are ligated together. Indels that create a shift in reading frame generate premature stop codons and loss of gene function, either through synthesis of truncated non-functional proteins or nonsense-mediated mRNA decay. Alternatively, co-delivery of Cas9 with multiple gRNAs targeting the same gene at different locations can generate multiple simultaneous DSBs resulting in deletion of large segments of intervening coding sequence, which completely inactivates the target gene (Cong et al., 2013).

Successful Knockout (KO) requires functional delivery of both the gRNA and Cas9 protein to the desired cell population. For *in vitro* experimentation, this can be achieved fairly readily by co-transfection of soluble RNAs (gRNA and Cas9 mRNA), preformed Cas9-gRNA RNPs, or dual expression cassettes on plasmid DNA or viral vector. Though most groups use expression cassettes for delivery, RNPs are more efficient with fewer off-target cuts. With careful gRNA sequence engineering, the Cas9 nuclease can be directed to selectively cleave the coding sequence (or regulatory region) of virtually any gene in nearly any location. While the procedure is fairly straightforward *in vitro*, careful screening is required at the DNA and protein level to identify and validate KO clones. For *in vivo* use viral vectors are required for Cas9-gRNA delivery to most tissues as transfection is too inefficient to generate a measurable response. Non-integrating, with low inflammatory potential, AAV is the preferred vector (Wang et al., 2020). The small genome can accommodate expression cassettes for Cas9 mRNA and up to two gRNAs (Bengtsson et al., 2017).

### Gene Knock-In

Precise genomic editing, e.g., targeted insertion of a fluorescent reporter, or the correction (or introduction) of a specific mutation, is achieved by inducing HDR and repair of Cas9-targeted DSBs by homologous recombination. In addition to delivery of the gRNA(s) and Cas9, an exogenous DNA template is also required that contains the desired sequences for insertion bracketed on either side by asymmetric sequence arms 50–800 bp in length, homologous to the genomic sequences on each side of the DSB. Recombination between the homologous regions of the genomic DNAs and corresponding sequence arms of the repair template inserts the template DNA cleanly into the host chromosome (Cong et al., 2013). Template DNA in the form of ssDNA oligonucleotide or linearized plasmid DNA is most effective. Due to the greater complexity, the generation of Knock-In (KI) models is substantially less efficient than KO. This situation is amplified *in vivo* where simultaneous co-infection of each target cell with two different AAV vectors is required- one for the CRISPR/Cas9 components and the second containing the template DNA (Wang et al., 2020; Bengtsson et al., 2017).

CRISPR/Cas9 technologies provide powerful tools for gene manipulation in studies of cell biology and differentiation (Yanagihara et al., 2019), disease mechanisms and signaling pathways (Suzuki et al., 2016; Lin et al., 2017; Zhao et al., 2019), and the roles of cancer-associated genes in tumorigenesis and drug resistance (Guernet et al., 2016; Marko et al., 2016; Xiao et al., 2018). By targeting germline cells of experimental animals, precise transgenic and KO models can be generated much more quickly than previous methods (Williams et al., 2018). However, many significant technical and biologic hurdles remain before gene editing can be considered for clinical use in orthopaedics (Cribbs and Perera, 2017; Lino et al., 2018). The low efficiency of KI modifications, off-target DNA cleavage and template insertion, immune recognition of bacterial Cas proteins, and long-term safety are all major concerns (Dai et al., 2016). The greatest obstacle, however, lies with delivery and the inability to transduce enough cells in tissues of human-scale to mediate a meaningful effect (Dai et al., 2016). This is especially problematic in orthopaedics (Fitzgerald, 2020), where the target tissues are ECM-dense and the predominant monogenic disorders are skeletal dysplasias (e.g., achondroplasia and osteogenesis imperfecta) that generate abnormalities throughout the entire skeletal system (Krakow and Rimoin, 2010; Costantini et al., 2021).

## *Ex Vivo* vs. *In Vivo* Delivery

Gene therapy in orthopaedics has primarily focused on the delivery and overexpression of cDNAs encoding cytokine inhibitors and growth factors, where the modified cells function as local factories for sustained production and release (Evans et al., 2021). Depending on the application, functional gene transfer can be mediated by *in vivo* (direct) or *ex vivo* (indirect) methods (Figure 1).

### *In Vivo* Gene Delivery

For gene delivery *in vivo*, a liquid suspension of vector is injected directly into the fluids or tissues of the host to modify accessible, receptive cell populations. The vector can be administered systemically (Mingozzi and High, 2013) or locally to sites of injury or disease (Evans et al., 2018b). Regarding systemic administration, counter to many reports, there is no mechanism by which recombinant vectors (viral or non-viral) (Pouton and Seymour, 2001), or modified cells (see below) delivered into the bloodstream can selectively target damaged or diseased skeletal/connective tissues and achieve functional expression. With limited exception, the genetic agents will be filtered or entrapped by the organs that process the blood (heart, liver, lungs, spleen, and kidneys). In orthopaedics, gene transfer is used to inhibit pathologic processes or augment repair at specific anatomic locations (skeletal fractures, arthritic joints, cartilage lesions etc.); and local gene delivery is the rational approach. By concentrating the vector and gene product at the site of need, the efficacious vector dose is substantially reduced, as is the risk of adverse off-target effects.

The advantages of *in vivo* gene transfer include: 1) clinical expediency, 2) straightforward, minimally invasive delivery, and 3) substantially reduced cost relative to protracted *ex vivo* procedures or cellular engineering strategies. Concerns over unwanted trafficking of vector to vital organs can be mitigated to a large degree by precise anatomic positioning of the injection needle via fluoroscopic, or ultra-sound guidance, the use of minimal injection volumes, and delivery at a controlled flow rate. Detailed pre-clinical studies of the biodistribution of vector genomes and modified cell populations in a relevant animal model, aid in the refinement of dosing and delivery procedures to minimize vector egress from the site of delivery (Watson Levings et al., 2018).

### *Ex Vivo* Gene Transfer

For *ex vivo* strategies, cells collected from the intended host are genetically modified in the laboratory, then returned to the donor to engraft and express the transgene product (Naldini, 2011; Dunbar et al., 2018). The modified cells can be injected in liquid suspension or seeded in biocompatible scaffolds for implantation. Depending on availability, the cell type selected for modification may be a differentiated resident of the target tissue or a multipotent cell from a distal location. Relative to *in vivo* delivery, *ex vivo* procedures offer additional layers of control and safety. No free vector is administered to the host, and the modified cells can be analyzed for quality, function, and the presence adventitious agents or RCR. In these respects, the practitioner controls the cell type, performance criteria, dose, and route of administration.

Despite these advantages, *ex vivo* procedures pose significant logistical hurdles that raise concerns of real-world utility for common orthopaedic disorders. Firstly, the number and quality of the cells recovered from individual donors can vary widely, as can growth rate, receptiveness to modification and transgene expression, particularly among older patients (Evans et al., 2005). Cells adapted to *in vitro* culture often die soon after implantation due to stress from the change in growth conditions. From a practical standpoint, *ex vivo* methods involve serial invasive procedures, are time-consuming, labor-intensive and require qualified GMP facilities, all of which elevate costs dramatically. In clinical trials, the costs of cell manufacturing alone range from $100,000 to $300,000 (or more) per patient (Elverum and Whitman, 2020). Although sophisticated genetic, cellular and tissue engineering strategies can be envisioned for numerous orthopaedic applications, time constraints and unfavorable cost/benefit will be prohibitive to clinical translation (Bara et al., 2016).

Recombinant lentivirus is the preferred vector for *ex vivo* procedures (Naldini, 2011; Dunbar et al., 2018). Stable insertion into the chromosomes of the modified cell, enables the expression cassette to be passed to both daughter cells following cell division. A few thousand cells can be transduced with a nominal amount of virus then expanded in culture to generate tens of millions (Naldini, 2011). Vectors whose genomes remain episomal (e.g., AAV, Ad, non-viral), can be used *ex vivo*, but are less than ideal. As extrachromosomal elements, the vector DNA will be progressively lost from dividing cell populations with each round of mitosis (Dunbar et al., 2018). To minimize the loss of transgene expression, the entire volume of cells required for delivery must be modified just prior to implantation. Efficient modification of large volumes of cells can be technically challenging and consume large quantities of clinical grade vector (Bara et al., 2016).

#### MSCs

Mesenchymal stromal cells (MSCs) are particularly well-suited to gene- and cell-based strategies for repair/regeneration of skeletal connective tissues (Bianco et al., 2008) especially those requiring supplemental cells to increase the cellularity and biosynthetic capacity of the damaged tissue. Although MSCs can be guided to differentiate into multiple mesenchymal lineages, the default pathway follows the chondro-osseous progression of endochondral ossification.

MSCs were first identified in the 1960’s as plastic-adherent, colony-forming unit fibroblasts (CFU-F) capable of differentiating into osteoblastic, chondrocytic and adipocytic lineages *in vitro* (Friedenstein et al., 1968; Friedenstein et al., 1970; Friedenstein et al., 1974). Relatively abundant in most mammalian tissues, MSCs are isolated most often from bone marrow and adipose tissue. Although numerous surface markers have been variously reported to enrich for MSC-like populations, The International Society for Cell and Gene Therapy has established the following standards for MSC designation: 1) adherence to plastic; 2) tri-lineage potential *in vitro* (osteoblastic, chondrocytic, and adipogenic differentiation), 3) expression of mesenchymal surface markers CD73, CD90, and CD105, and 4) the absence of characteristic hematopoietic markers (e.g., CD45, CD31, and CD14) (Dominici et al., 2006). By these broad criteria, MSCs are functionally and phenotypically indistinguishable from the fibroblasts that produce collagenous support matrix in virtually every tissue in the body (Haniffa et al., 2009; Prockop, 2009; Ugurlu and Karaoz, 2020).

In 1991, the re-branding of the CFU-F as a “mesenchymal stem cell,” (Caplan, 1991) together with aggressive promotion and marketing, served to elevate the perception of MSCs among the research community and lay public to the current status of miracle cure-all (Sipp et al., 2017; Sipp et al., 2018). According to the published literature, irrespective of the disease model or its pathogenesis the administration (by any route) of an arbitrary dose of MSCs from virtually any species, tissue, or culture protocol, will induce a pronounced therapeutic response, (Caplan, 2017; Sipp et al., 2017; Sipp et al., 2018). Following systemic delivery, MSCs are reported to selectively migrate to the diseased tissue of investigator interest to halt ongoing pathologies and mediate repair, even when only briefly present at homeopathic levels (Prockop, 2009). In culture, MSCs are reported to secrete regenerative growth factors and immune suppressive molecules capable of restoring full function to all diseased or damaged tissues (somatic or germ line), enable allogenic transplantation and inhibit autoimmune disease.

The MSC secretome, however, is an artifact of *in vitro* culture and reflects adaptation to growth in monolayer in synthetic medium enriched with fetal protein factors and various cytokine cocktails (Brooks et al., 2019). Despite numerous claims, MSCs are not immune-privileged. Following delivery in immune competent hosts, allogenic MHC-mismatched MSCs elicit potent humoral and CTL responses (Nauta et al., 2006; Poncelet et al., 2007; Zangi et al., 2009; Ankrum et al., 2014; Berglund et al., 2017; Kamm et al., 2020). When administered systemically MSCs do not home to bone marrow or sites of disease or injury at appreciable levels, but instead become trapped in the organs that process the blood (lungs, liver, heart, spleen, and kidney), such that the vast majority die within 48 h of injection (Lee et al., 2009; Prockop, 2009; Makela et al., 2015; Masterson et al., 2021).

Setting aside the implausible medicinal properties, the controversies, inconsistencies and disagreements that surround the MSC field (Prockop, 2009), the one area of universal agreement is the ability of MSCs to differentiate into chondrocytic and osteoblastic phenotypes and elaborate the corresponding ECM components. This multipotency is reflected *in vivo* in a variety of pathologic metaplastic conditions: e.g., the formation of ectopic chondro-osseous nodes in synovial chondromatosis, osseous metaplasia in the endometrium and gastrointestinal tract and heterotopic ossification of muscle and tendon following injury. Thus, for skeletal/connective tissue repair, MSCs can fulfill two important roles: 1) as genetically engineered factories for prolonged synthesis and release of bioactive gene products, and 2) as a readily available cell source capable of adopting different mesenchymal phenotypes and contributing to the synthesis and maintenance of repair tissues. Clinical translation, however, will require methods that support and preserve directed differentiation and retain the modified cells at the site of implantation in a viable functional state. For each experimental application detailed tracking studies are required (along with objective, unbiased reporting) to firmly establish the temporal fate of the transplanted MSCs, their density, distribution, lifespan, and contributions, if any, to the generation of repair tissues.

#### Induced Pluripotent Stem Cells

Induced pluripotent stem (IPS) cells offer a source of pluripotent cells with promise in *ex vivo* gene therapy applications. The transient delivery and expression of cDNAs for a cocktail of transcription factors that regulate pluripotency and cell division (OCT4, KLF4, NANOG, and MYC; *OKNM*) induces wholesale epigenomic reprogramming to render terminally differentiated cells into a primordial pluripotent state (Takahashi and Yamanaka, 2006; Takahashi et al., 2007; Wernig et al., 2007). Once reprogrammed, IPS cells can be directed to differentiate along any lineage to provide a boundless supply of donor-autologous cells of any desired phenotype for regenerative applications or gene correction strategies (Tsumaki et al., 2015).

IPS technology has been used to reprogram and transdifferentiate fibroblasts into a variety of mesenchymal cell types, including chondrocytes (Craft et al., 2015; Iimori et al., 2021), osteoblasts (Tashiro et al., 2009; Li et al., 2010; Bilousova et al., 2011), tenocytes (Komura et al., 2020; Nakajima et al., 2021) and anulus pulposus cells (Chen et al., 2013; Tang et al., 2018), whose phenotypes appear stable *in vivo*. While IPS cells appear promising for orthopaedic applications, reprogramming efficiency and cellular phenotype post-differentiation still remain highly variable. IPS cells routinely form teratomas following implantation *in vivo*, which brings concerns of tumor formation from undifferentiated subpopulations (Yamanaka, 2020). Further, the extensive handling and manipulation required for reprogramming, modification, expansion, and re-differentiation currently render this technology impractical for routine clinical use. Thus, for the foreseeable future, IPS cells will likely remain as experimental tools.

## Experimental Models

While *in vitro* assays are useful for assessment of vector function and transgene expression, the results are not representative of the efficacy of gene delivery *in vivo*. With limited exception, cells in culture are far more receptive to genetic modification than in living tissues. They divide rapidly and arrayed on two-dimensional surfaces free of ECM, provide maximum surface area and availability to recombinant vectors.

**FIGURE 4:**
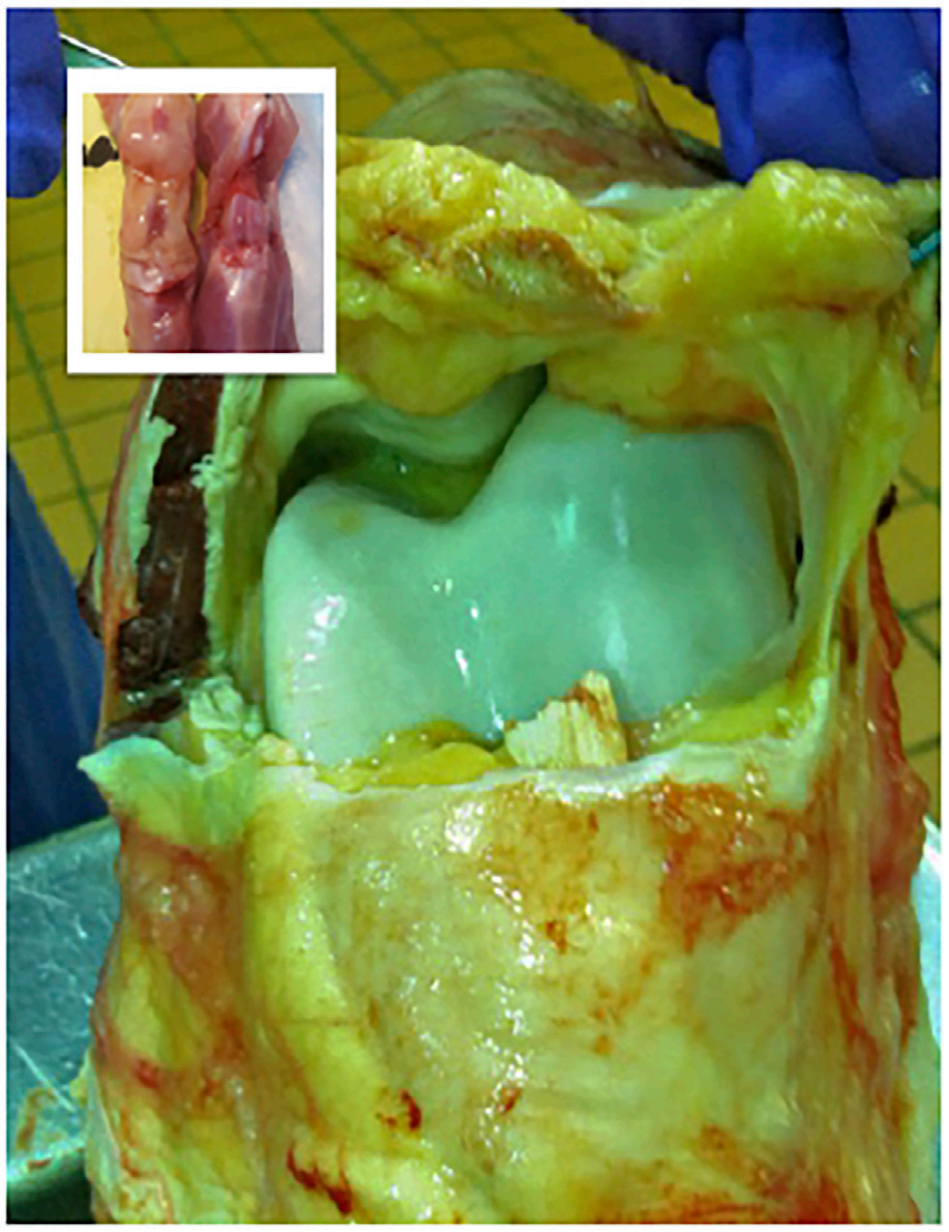
Comparison of the internal anatomic structures of the stifle joints (hind knees) of the adult rat (inset) and the horse, which is comparable in size to the human knee. The striking differences in magnitude, volume and thickness of the tissues underscore the practical challenges associated with the scale-up of a gene-based therapy to a large mammalian system.

Therapeutic gene transfer is an extraordinarily complex process that can only be approximated in the context of an immune-competent animal and relevant disease model. When attempting to treat a condition with molecular tools it’s vital that the pathogenesis and pathology of the experimental model reflect the human condition as closely as possible at the organ, tissue, cellular and molecular levels. Common laboratory animals (e.g., mice, rats, rabbits) are useful for proof-of-concept studies and exploration of basic methodology. Small animals, though, have a remarkable capacity for self-repair and frequently exaggerate the efficacy of regenerative strategies and the facility with which they can be performed. In these respects, large animal models (e.g., sheep, horses, goats, pigs, cows) are essential to the clinical advancement of orthopaedic gene therapies. With skeletal tissues comparable in size to those of humans, with similar thickness and architecture (Figure 4), experimentation in appropriate large animal systems provides information regarding vector dosing, transgene expression, biodistribution and efficacy, directly relevant to clinical application. As experimental therapies in orthopaedics typically involve surgical application, large animals better depict the logistics, ergonomics, and efficacy of the procedure in a clinical setting. Since most large animals are outbred, they more closely reflect the genetic and phenotypic diversity of the human population.

## Experimental Progress

### Rheumatoid Arthritis

Orthopaedic gene therapy was conceived in the early 1990’s to tap into the therapeutic potential of a growing list of proteins with promising activities for RA (Bandara et al., 1993; Evans and Robbins, 1995b). The inherent instability of recombinant proteins *in vivo*, coupled with the continuous turnover of synovial fluid in diarthrodial joints, provided only transient effects following intra-articular injection. Further, as many of the candidate proteins suppressed inflammation and cytokine signaling, elevated levels in the circulation brought the potential for systemic immune suppression and vulnerability to infection. By delivering the cDNAs to cells in the articular tissues the gene products could be continuously produced to block ongoing erosive pathologies (Evans and Robbins, 1995b).

Limited by available vector technology, an *ex vivo* procedure was employed involving transduction of autologous synovial fibroblasts with a γ-retroviral vector (MFG) containing the cDNA for interleukin-1 receptor antagonist (IL-1Ra: an inhibitor of IL-1 signaling) (Bandara et al., 1993; Boggs et al., 1995). Following transduction, expansion, and analysis for IL-1Ra expression, sterility and RCR, the cells were injected into RA joints of the respective donors to engraft and express the IL-1Ra transgene. Pre-clinical studies (Bandara et al., 1993; Evans and Robbins, 1995a) followed later by successful phase I trial (Evans et al., 1996; Evans et al., 2005), showed that intra-articular gene delivery was feasible and safe. Unfortunately, the logistics and expense made the procedure impractical for mainstream use.

### Osteoarthritis

With the development of recombinant tumor necrosis factor (TNF) receptors and anti-TNF antibodies (e.g., etanercept and adalimumab, respectively), which proved effective in a majority of RA patients, the focus of arthritis gene therapy shifted to OA (Evans et al., 2004). While RA is a systemic polyarticular autoimmune disease, OA is a progressive degenerative condition that affects one or two joints per patient and has no known extra-articular component (Goldring and Berenbaum, 2015; Martel-Pelletier et al., 2016). Existing OA medications reduce joint pain but have no effect on joint degeneration (Martel-Pelletier et al., 2016).

To make gene transfer more applicable and cost-effective, direct intra-articular injection of candidate vectors has been extensively explored (Roessler et al., 1993). Patterns of intra-articular transgene expression have been characterized from every well-developed vector system available, viral, and non-viral (Nita et al., 1996; Evans et al., 2021). Vectors delivered intra-articularly diffuse through the synovial fluid and interact primarily with the synovium, due to its large surface area and high cellularity. Without a basement membrane separating the intimal fibroblasts from the joint fluids, the abundant synovial fibroblasts are immediately available and receptive to modification from most viral vectors (Nita et al., 1996; Evans et al., 2021). Temporal quantification of transgene products in synovial fluid reveals the efficiency of gene delivery and cumulative transgene expression and its persistence over time (Bandara et al., 1993; Evans et al., 1999). With the use of homologous transgenes and vectors with a low immunogenic profile, articular cells are capable of supporting robust transgene expression for over a year (Gouze et al., 2007; Goodrich et al., 2013; Goodrich et al., 2015; Watson Levings et al., 2018).

**FIGURE 5:**
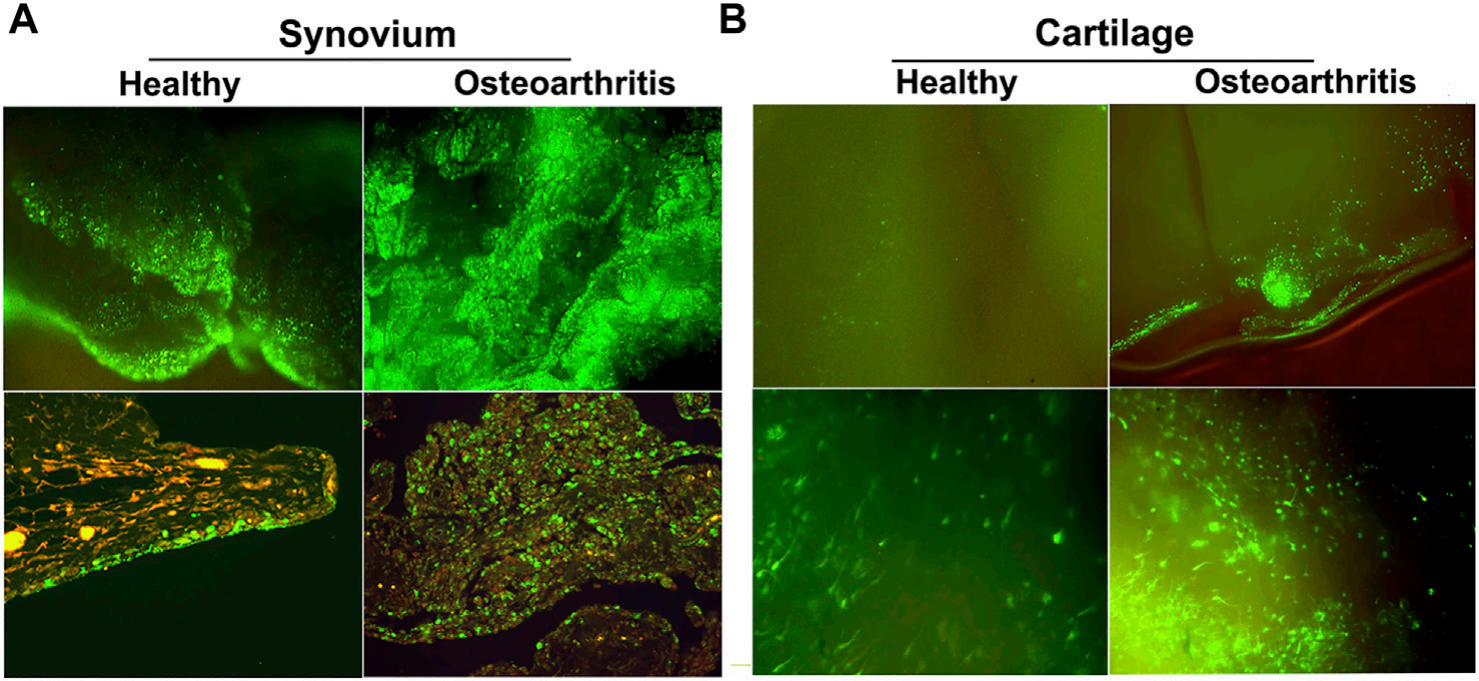
GFP expression in healthy and OA joints following intra-articular gene delivery with scAAV. The middle carpal joints of 3 healthy horses and 3 with late stage naturally-occurring OA were injected with 5 × 10^12^ vg of scAAV.GFP. Two weeks later the joint tissues were collected and analyzed for fluorescence. **(A)** (Top row) Fluorescence activity in freshly harvested synovial tissues viewed with inverted fluorescence microscopy at ×10 magnification. (bottom row) Paraffin sections of synovium immunohistochemically stained for GFP at ×20 magnification. In normal joints, the synovium was the predominant site of transgene expression, with abundant fluorescent cells scattered throughout the capsular lining, often concentrated in thicker villous regions. In striking contrast, the number and density of the fluorescent cells in OA joints were visibly greater across the entire expanse of the synovial lining, but particularly so in regions with marked hyperplasia and leukocytic infiltration. In both normal and OA joints the transduced cells were almost exclusively delimited to the synovium and subsynovium, and only rarely seen in the supporting fibrous tissues. **(B)** GFP expression in fresh cartilage shavings viewed with inverted fluorescence microscopy. Images in the top and bottom rows are at ×10 and ×20 magnification, respectively. In articular cartilage from normal joints, GFP fluorescence was visible but generally faint and limited to scattered isolated cells. In OA cartilage, GFP activity was dramatically enhanced, as populations of brightly fluorescent cells were readily apparent in all shavings recovered. The labeled chondrocytes included both elongated cells, consistent with superficial layer chondrocytes, and cells with more spherical morphology characteristic of chondrocytes in deeper layers. Shavings harvested near full thickness erosions often contained focal regions with intense fluorescence readily visible at low magnification.

Of the available vector systems, rAAV is currently the most promising for use in OA, offering efficient gene delivery with a safety profile compatible with treatment of common, non-life-threatening conditions. Following injection in the joints of large animal models, rAAV can modify synovial fibroblasts and chondrocytes in articular cartilage with high efficiency to provide expression of a homologous therapeutic gene product at levels 50 to 100x over endogenous production. Due to its uniquely small size (∼20 nm dia.) AAV is the only vector system capable of penetrating the cartilage ECM to modify articular chondrocytes *in situ* (Watson Levings et al., 2018). This is particularly valuable in OA since cartilage degradation is the characteristic pathology, and the chondrocytes are responsible for maintaining cartilage matrix homeostasis (Figure 5).

A wide range of gene products have been tested in experimental OA and reported to provide benefit. IL-1Ra has been used most frequently, and has consistently shown marked anti-inflammatory and chondroprotective effects (Evans et al., 2021). Currently two clinical trials of OA gene therapy are listed as active with clinicaltrials.gov (https://clinicaltrials.gov/ct2/home). Both involve direct *in vivo* delivery of IL-1Ra cDNA; the first via scAAV (NCT02790723), while the second employs HC adenovirus (NCT04119687).

**FIGURE 6:**
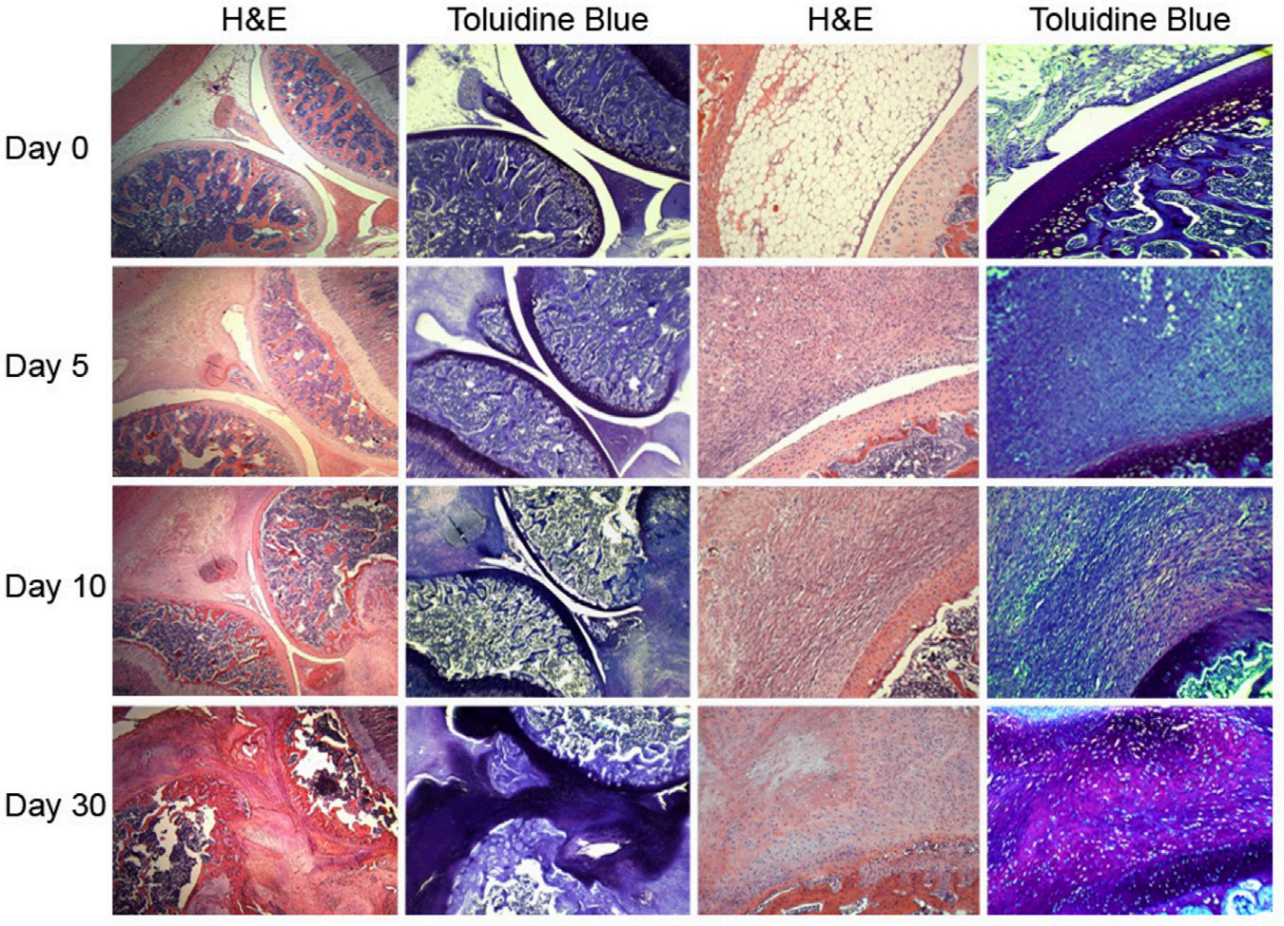
Severe synovial fibrosis and chondrometaplasia induced by intra-articular gene delivery of TGF-β1. The stifle joints (hind knees) of nude rats were injected bilaterally with 2 × 10^9^ viral particles of Ad.TGF-β1. Groups of animals were killed at days 0, 5, 10, and 30, and the joints were harvested and processed for histology. Adjacent sections were stained with H&E or toluidine blue as indicated. The images in left two columns are at ×2.5 magnification, while those in the right two columns are at ×20. At Day 5, expansion of spindled fibroblasts from the synovial lining and joint capsule produced a dense fibrotic mass that fully occluded the underlying adipose layer. By Day 10, the fibrotic tissue had expanded to displace all the soft tissue structures and began to fuse with articular cartilage, in which the development of rounded chondrocytic cells can be seen. By Day 30, the bulk of the fibrotic expanse had differentiated into a cartilaginous phenotype as indicated by the cellular morphology and metachromatic toluidine blue staining. In some areas the normal articular cartilage was replaced by metaplastic fibrocartilaginous tissue that also permeated the subchondral and periarticular bone. Figure copied with permission from (Watson et al., 2010).

A third trial whose status is listed as “unknown” describes an *ex vivo* approach involving intra-articular injection of irradiated allogenic chondrocytes modified with a γ-retroviral vector to express TGF-β1, mixed 1:3 with unmodified irradiated cells (NCT03383471) (Evans et al., 2018a). The conceptual basis for this trial is of particular concern. Several independent tracking studies have shown that cells injected into the joint do not adhere to cartilage surfaces, damaged or otherwise, but primarily engraft in the synovial lining, and to a lesser extent the surface of the meniscus (Murphy et al., 2003; Desando et al., 2013; Grady et al., 2019; Enomoto et al., 2020). Further, overexpression of TGF-β1 intra-articularly induces severe synovial fibrosis (Figure 6) (Watson et al., 2010), and in immune competent hosts, allogenic cells are recognized as foreign and are killed by CD8^+^ T cells (Nauta et al., 2006; Poncelet et al., 2007; Ankrum et al., 2014; Cao et al., 2016). Analysis of the modified cells used in clinical trials in South Korea and the U.S. revealed that they were not in fact chondrocytes, but instead were 293 cells. While initial approval in South Korea has been revoked; oddly, the once-suspended phase III study in the US has been allowed to resume. The extent to which this protocol will go forward is unclear.

### Cartilage Repair

ECM-dense, avascular with sparse cellularity, articular cartilage has minimal capacity for self-repair. In injuries limited to the chondral tissues, there is no rupture of blood vessels or influx of fluids, proteins, or cells to fill the void of the lesion or initiate space-filling repair (Ghivizzani et al., 2000). With no natural guide to inform reparative strategies, clinical approaches attempt to create functional repair substances *de novo* (Ghivizzani et al., 2000). These procedures generally result in the formation of fibrocartilaginous tissues that offer acceptable levels of improvement, at least in the short-term. Gene-based strategies look to augment the efficacy and durability of these and related procedures, to generate repair tissue with the phenotype of hyaline cartilage and fully restore joint function.

Several growth factors from the TGF-β superfamily are known to induce and sustain chondrogenic differentiation and matrix synthesis in mesenchymal cells but require prolonged delivery (14–21 days) at high levels (Bobick et al., 2009). By modifying target cells to express and secrete these factors as transgene products (Palmer et al., 2005), prolonged growth factor delivery and effective chondrogenesis can be achieved *in vitro* and *in vivo* (Graceffa et al., 2018). Though numerous reports describe repair strategies and cDNAs that improve cartilage healing, the best platform for long-term repair remains elusive (Trippel et al., 2007; Bougioukli et al., 2018a).

Due to the inability of exogenous cells to adhere to and colonize cartilage surfaces, and the inability of resident chondrocytes to migrate to sites of damage (Desando et al., 2013; Grady et al., 2019; Enomoto et al., 2020), there is no logical mechanism by which the intra-articular injection of modified chondrocytes or MSCs in suspension can mediate space-filling repair of cartilage lesions or regenerate cartilage surfaces in OA. Fibroblasts resident in the synovium have potent MSC-like properties and are highly responsive to growth factor stimulation. Elevated levels of anabolic factors in the articular tissues and fluids stimulate the fibrotic expansion of the synovial lining that progressively undergoes chondro-ossification (Figure 6) (Watson et al., 2010). To avoid the induction of synovial chondrometaplasia, gene delivery strategies are required that delimit growth factor production and signaling specifically within cartilage lesions (Pascher et al., 2004b).

*Ex vivo* repair methods initially focused on the implantation of genetically modified chondrocytes to enhance the efficacy of autologous chondrocyte transplantation (ACI) (Palmer et al., 2005; Koga et al., 2008). Implantation into cartilage defects of chondrocytes modified to express growth factors FGF-2, IGF-1 or BMP-7 either alone or in combination has been reported to stimulate cartilage ECM production *in vitro* and enhance the volume and quality of cartilaginous repair tissue in laboratory animals as well as in horses (Hidaka et al., 2003; Trippel et al., 2004; Shi et al., 2012; Caldwell and Wang, 2015; Ortved et al., 2015; Griffin et al., 2016).

While the use of autologous chondrocytes for repair applications is preferrable, the supply of cartilage available for cell harvest is limited and its use requires two invasive procedures. These technical constraints have driven many in the cartilage repair field to focus on MSCs. Though more abundant and accessible than chondrocytes, MSCs require differentiation into chondrocytes and possibly sustained growth factor stimulation to maintain the phenotype. MSCs from a variety of tissues (Ying et al., 2018) modified to express cDNAs for IGF-1, TGF-β1, BMP-2, and SOX-9 both alone and in combination have been reported to generate repair tissues enriched in collagen II and proteoglycan with the histologic phenotype of articular cartilage. Due to the lack of tracking studies, however, the extent to which the modified cells participate in the repair process or contribute to the cartilaginous repair tissue is uncertain.

Marrow stimulation techniques, such as microfracture, create channels to allow the upwelling of progenitor cells from the marrow where they acquire a fibro-chondrocytic phenotype and elaborate ECM to restore cartilage volume. Methods to genetically modify the marrow cells in situ as they immigrate into the cartilage lesion have been explored. The most direct approach involves the deposition of a small volume of rAAV directly into the open microfracture (Cucchiarini et al., 2018). Alternatively, a vector suspension can be mixed with fresh bone marrow aspirate and following coagulation press-fit the into the osteochondral lesion to form a “gene plug.” Following feasibility studies with Ad vectors containing GFP and luciferase reporters (Pascher et al., 2004a), the implantation of marrow coagulates expressing TGF-β1 in partial thickness chondral lesions generated in sheep provided enhanced repair relative to unmodified marrow clots and significantly increased collagen II levels (Ivkovic et al., 2010). More recently in comparisons of cartilage repair following delivery of marrow coagulates expressing Indian Hedgehog (IHH) or BMP-2 into osteochondral lesions in rabbits (Sieker et al., 2015), sustained BMP-2 expression was associated with formation of osteogenic foci in the repair tissue, while expression of IHH induced a more hyaline repair phenotype. The strengths of the gene plug procedure lie in its simplicity and practicality; efficacy, though, relies on the cellularity of the bone marrow sample at the time of withdrawal, which can vary considerably among aspirates and individuals (Sieker et al., 2015).

### Bone

Distinct from other connective tissues, bone has robust capacity for self-repair. With appropriate stabilization, endogenous mechanisms can fully restore the structure and function of most closed fractures. When clinical need exceeds the repair capabilities of the patient, surgical intervention is required to facilitate healing. In this regard, certain co-morbid conditions, e.g., diabetes (Gandhi et al., 2006), aging (Gruber et al., 2006), osteoporosis (Giannoudis et al., 2007), smoking (Kwiatkowski et al., 1996), etc. can predispose individuals to skeletal fracture and contribute to deficient repair and atrophic non-unions. Compound open fractures or loss of significant bone volume can generate lesions that exceed the regenerative capacity of even healthy individuals. Spinal fusion and implant fixation present additional scenarios where the capacity to augment local bone synthesis could improve clinical outcome.

A diverse range of biomaterials is available to augment surgical repair of skeletal lesions (Laurencin et al., 2006; Fillingham and Jacobs, 2016; Morris et al., 2018); each has certain benefits and limitations. Bone autograft, harvested from the iliac crest, is the clinical standard. Variously comprised of marrow, progenitor cells, osteoblasts, osteocytes and bone matrix, the graft materials readily assimilate and synthesize bony repair tissue. The volume of tissue available for harvest can be limiting, and collection is invasive, often painful, with the risk of complications. The reamer irrigator aspirator technique enables procurement of bone matrix and marrow from an intact femur (Porter et al., 2009), but is also invasive and risks damaging healthy bone. Autograft materials can be implanted alone or combined with devitalized bone allograft or synthetic ceramic scaffolds that provide structure and physical support but lack cellularity and osteoinductive properties. As allograft bone does not revitalize or remodel, it can become brittle with a high incidence of failure. Demineralized bone matrix (DBM; the acid-extracted organic matrix of human bone) is approved for use in large-scale skeletal lesions and spinal fusions (Gruskin et al., 2012). Though its osteoconductive and osteoinductive properties are well-established, variable composition, even in pharmaceutical grade DBM, can make it difficult to apply predictably (Gruskin et al., 2012; Glowacki, 2015).

Initial cloning of the osteoinductive factors in DBM (Urist, 1965), the “bone morphogenetic proteins” (BMPs) -1, -2, -4, and -7 revealed a novel family of osteoinductive ligands related to the TGF-β superfamily (Lyons and Rosen, 2019). Since then, a total of 15 BMP isoforms have been identified with overlapping pleiotropic activities. Among these, BMPs-2, -4, -6, -7, and -9 (Fujioka-Kobayashi et al., 2018) have the greatest chondrogenic/osteoinductive activity *in vivo*. Recombinant human (rh)BMP-2 and BMP-7/osteogenic protein 1 (OP1) have been studied extensively and display potent osteogenic activity (Heckman et al., 1991; Gerhart et al., 1993; Lee et al., 1994; Bostrom and Camacho, 1998). Both are FDA-approved for use in large skeletal defects and spine fusions. Initially high enthusiasm over the use of rhBMPs in orthopaedics has since dimmed (Glowacki, 2015) due to their propensity for adverse side effects, e.g., infection, edema, swelling, heterotopic bone formation, and neural complications, especially in spinal fusions (Shields et al., 2006; Smucker et al., 2006; Perri et al., 2007; Benglis et al., 2008; Hsu and Wang, 2008).

Short half-lives and reduced activity *in vivo*, make recombinant proteins difficult to administer clinically for bone repair, requiring extremely high doses (with high costs) for prolonged effect. The milligram quantities of rhBMP-2 used in spinal fusions are estimated to be ∼1,000,000 times greater than naturally exists in the adult skeleton (Hsu and Wang, 2008; Glowacki, 2015). Delivered in a single massive bolus in a hydrated collagen sponge (Barnes et al., 2005), osteoinductive protein often diffuses from the implant site to stimulate ectopic bone synthesis in neighboring tissues. Gene transfer provides an alternate delivery approach that may be more favorable and economical. By delivering the cDNAs for osteoinductive growth factors to cells within the repair milieu, prolonged stimulation can be achieved in a more physiologic range. Relative to rhBMP-2 produced in bacteria, hBMP-2 expressed as a transgene product by mammalian cells is processed, glycosylated, and transmitted to neighboring cell populations in a more natural form and context, providing enhanced bioactivity at 100 to 1000-fold lower concentrations (Bessho et al., 1999; De la Vega et al., 2021).

Following initial demonstrations that local gene delivery methods could be used to induce ectopic bone deposition in rodents (Boden et al., 1998; Lieberman et al., 1998; Kang et al., 2004), numerous gene delivery strategies involving both direct and *ex vivo* approaches have been reported to stimulate osteogenesis and bone repair *in vivo* (Bez et al., 2020; De la Vega et al., 2021). Direct gene delivery of an adenoviral vector containing the cDNA human BMP-2 into an open fracture model was found to provide robust transgene expression that peaked around 2 weeks post-delivery and then declined during weeks 3–6. Ad-mediated BMP-2 expression was of sufficient amplitude and duration to heal critical sized defects in rabbits (Baltzer et al., 2000) and in a fracture model in sheep with experimental osteoporosis (Egermann et al., 2006a).

Bone repair/regeneration is a multi-step process that begins with an inflammatory phase to establish hemostasis and activate phagocytic cells to remove debris and microbes. Local angiogenesis followed by an influx of mesenchymal progenitors drives the formation of a provisional chondro-osseous matrix that’s subsequently remodeled into mineralized bone. Throughout this process, the coordinate expression of multiple signaling molecules and growth factors is induced at different stages (Aono et al., 1995; Israel et al., 1996; Cho et al., 2002; Fujimura et al., 2002). In line with this, combinatorial delivery of Ad vectors containing cDNAs for BMP-2, BMP-4, and BMP-7 and their expression as heterodimers was found to increase osteoblastogenesis and enhance fracture repair and spinal fusion in rats relative to either alone (Zhu et al., 2004; Zhao et al., 2005; Kaito et al., 2013). As blood flow and oxygenation are essential for osteogenesis, gene delivery of angiogenic factors, such as VEGF (Peng et al., 2002) and cyclooxygenase 2 (COX-2) (Lau et al., 2013) has also been found to enhance bone synthesis *in vivo*. Similarly gene delivery of other growth factors, including Nell-1 (Aghaloo et al., 2007), IGF-1, TGFβ-1 have been reported to stimulate bone formation *in vivo*, as well as *ex vivo* delivery of osteogenic transcription factors RUNX-2 and Osterix, *Ex vivo* strategies employing genetically modified MSCs have the potential to enhance bone repair from two directions (Baltzer et al., 1999; Lieberman et al., 1999; Musgrave et al., 2000). First, the modified MSCs serve as local factories for prolonged synthesis and secretion of osteoinductive gene products. Secondly, they provide an exogenous supply of osteoprogenitor cells receptive to autocrine stimulation, to expand the regenerative, osteogenic capacity of the endogenous repair milieu in defects with substantial loss of bone volume. Most well-characterized viral vector systems are capable of modifying MSC preparations with reasonable efficiency (Virk et al., 2008). Relative to Ad vectors which provide a transient high-level burst of BMP expression, LV.BMP-2 expression in MSCs is lower but more stable and produces greater volumes of ectopic bone in SCID mice (Sugiyama et al., 2005). Intuitively, bone marrow-derived MSCs (Chan et al., 2018; Gulati et al., 2018), would appear to have the greatest osteogenic potential; however, in certain applications MSCs from human adipose tissue have been noted to perform comparably (Bougioukli et al., 2018b; Vakhshori et al., 2020).

Given the diversity of the human population and the range of skeletal injuries, gene-enhanced repair will require integration of assorted biologic components into individualized paradigms. Lieberman et al. have examined LV.BMP-2 expression and bone repair using MSCs from disparate human donors. Following transduction and quantification of BMP-2 production, varying doses of modified cells from each donor were deposited onto support matrices and implanted into segmental bone lesions in rats (Ihn et al., 2021). Dose dependent bridging repair was observed in all cases, though higher doses of modified cells also increased the incidence heterotopic ossification. Importantly, these studies showed that MSCs from young and middle-aged donors are similarly effective in gene-enhanced bone repair (Kang et al., 2021). More recently customized 3D printed hydroxyapatite β-tricalcium phosphate (TCP) scaffolds were shown to provide effective delivery of LV.BMP-2 modified MSCs and enhanced repair of critical sized bone defects (Alluri et al., 2019; Kang et al., 2021) demonstrating the feasibility of using cell-seeded scaffolds customized to the dimensions of the individual.

While the implantation of exogenous cells and support matrices can facilitate repair of large skeletal defects, lengthy *ex vivo* procedures are ill-suited to acute injuries (Evans et al., 2007). In this vein, abbreviated/expedited methods are in development that are more cost-effective and applicable for routine use. The first involves an abridged protocol for lentiviral transduction of autologous bone marrow cells, whereby the standard multiweek process of harvest, transduction, expansion and delivery is condensed to a one (Virk et al., 2011) or two-day (Bougioukli et al., 2019) procedure, applicable to pre-planned operative procedures and acute skeletal trauma. Following bone marrow aspiration, the buffy-coat layer containing the MSC fraction is incubated with LV.BMP-2 for 1 h prior to intra-operative implantation in a stabilized defect (“same day” delivery). A “next-day” variation allows overnight LV transduction to enhance BMP-2 expression. In a rat femoral defect model, both procedures provided effective bridging repair, but the “next day” procedure provided greater consistency (Alaee et al., 2015).

The second method involves the implantation of autologous muscle or adipose tissue modified to overexpress osteoinductive factors. Readily accessible in large quantities both tissues provide natural three-dimensional support matrices, pre-loaded with MSCs. Muscle, in particular, is rich with osteogenic cells that directly and substantively contribute to the repair of adjacent fractures (Julien et al., 2021). For this procedure, an appropriate volume of muscle or fat is biopsied from the anesthetized donor; divided into small pieces to enhance diffusion, then incubated with Ad.BMP-2 in the operating room. Afterward, the modified tissue segments are implanted into stabilized bone lesions in the same operative session. While Ad.BMP-2 modified grafts from both muscle and fat (Evans et al., 2009; Betz et al., 2010) mediated bridging repair of femoral defects, muscle provided more robust bone formation and greater consistency (Betz et al., 2015; Betz et al., 2016). As a prelude to large animal studies (Liu et al., 2015), Ad.BMP-2 modified muscle grafts from sheep were found to induce bridging repair in femoral defects of immune compromised rats. Importantly, mechanistic tracking studies using Ad.BMP-2 modified muscle grafts from transgenic GFP + donors demonstrated that the interstitial cells in the graft, secreted transgenic BMP-2 and differentiated into chondro-osseous cells in the endochondral repair milieu, and stably contributed to the bony repair tissue. These findings show a direct functional contribution of exogenous, genetically modified MSC-like cells in tissue healing and repair. This efficient, technically straightforward approach simultaneously solves multiple challenges that confound tissue engineering strategies in skeletal repair (De la Vega et al., 2020; De la Vega et al., 2021).

As with gene-based therapies in general, the induction of CTL responses targeted to the modified cell populations can significantly impede efficacy (Egermann et al., 2006b). In some cases, rapid, high-level induction of transgene expression can stimulate repair responses that outpace the emergence of antigen-targeted CTLs. Immune reactivity varies with species, anatomic location, transgene, as well as vector quality and purity. In studies of bone repair, the cDNAs used most frequently are of human origin, and amino acid (AA) sequence conservation can vary widely among animal species. For example, the AA sequence of human BMP-2 is 100% identical to that of the rat, but only shares 95.7% identity with the rabbit orthologue and 93.7% similarity with sheep. Due to varying levels of immunogenicity among different species, gene delivery of human BMP-2 can produce inconsistent results in these and other model systems. In some circumstances, immune-mediated failure can be averted by transient immune suppression with pharmaceuticals, such as FK506 (De la Vega et al., 2020; De la Vega et al., 2021). Encapsulation of immunogenic cells in hydrogel microspheres sufficiently permeable to allow diffusion of BMP-2 (or other gene products), but sufficiently dense to shield the cells from immune surveillance was found to prevent CTL activation to allow prolonged survival and transgene expression (Sonnet et al., 2013). Effective immune suppression or shielding of allogenic cells could streamline *ex vivo* gene delivery dramatically, to provide an off-the-shelf cell source with validated osteoinductive activity available for use in cases of acute trauma.

### Intervertebral Disc

The intervertebral discs (IVD) cushion and protect the vertebral bodies from axial compression and provide the spine with increased flexibility and stability. As the largest avascular tissue, the IVD is principally comprised of fibrocartilaginous ECM, maintained by sparse populations of chondrocytic and fibroblastic cells. The IVD normally undergoes age-related changes in morphology and composition that increase with advancing years (Boos et al., 2002). IVD degeneration (IVDD), however, is a distinct pathologic condition involving age-accelerated tissue degradation leading to structural failure (Dowdell et al., 2017). Though its etiology is poorly understood, IVDD is widely attributed to the interplay of genetic predisposition and environmental factors (Wong et al., 2019). Progressive loss of ECM structure and cellularity alters the disc mechanics causing overloading, deformation and persistent, often debilitating, pain (Sakai and Grad, 2015).

The core of the IVD, the nucleus pulposus (NP) is comprised of type II collagen and elastin fibers that enmesh a hydrated gelatinous matrix of aggrecan. The matrix is populated at low density with cells of notochordal or chondrocytic phenotype. The cushioning capacity of the IVD stems from the bottle-brush structure and electrostatic charge of the glycosaminoglycan chains bound to the aggrecan core protein, which imbibe water and constrain its flow under mechanical pressure (Dowdell et al., 2017). The anulus fibrosus (AF) surrounds the NP, forming a fibrous boundary of concentric lamellar bands of collagens I and III that prevent its deformation under loading. The thin cartilaginous end plates (CEPs) above and below the NP and AF, are composed of hyaline-like cartilage and bind the fibrocartilaginous disc to the bony end plates of the adjacent vertebrae. Nutrition is provided to the avascular IVD by diffusion from capillary beds in adjacent vertebral bodies. Furthest from the blood supply, the NP cells are adapted to anaerobic metabolism, creating a hypoxic environment high in lactate with low pH (Sakai and Grad, 2015).

IVDD pathogenesis reflects declines in vertebral vascularity (Dowdell et al., 2017) and CEP permeability (Boos et al., 2002; Wong et al., 2019) that increasingly limit oxygen and nutrient availability. Metabolic stress in the IVD cells (Kadow et al., 2015) coupled with inflammatory signaling and/or oxidative stresses from environmental factors (e.g., smoking, obesity, genetic polymorphism etc.) induces production of MMPs, aggrecanases, proteolytic enzymes, and pro-inflammatory cytokines that disrupt ECM homeostasis in favor of catabolism (Dowdell et al., 2017). In the NP, the loss of proteoglycan content and increased deposition of collagen fibers increases its stiffness and diminishes hydraulic cushioning (Kadow et al., 2015). As the IVD loses height and clefts begin to form increased physical stress causes the NP cells to adopt a fibroblastic phenotype and increase production of inflammatory mediators and MMPs. Regional cell populations begin to senesce or die from apoptosis and necrosis. The AF undergoes similar degradative changes, including proteoglycan loss and dehydration. The collagen fibers become disorganized, stiffer, and weaker, and the collagen-elastin network gradually deteriorates. As fissures begin to form, leakage of inflammatory cytokines and degenerative byproducts stimulates vascular and neural ingress (Kadow et al., 2015), contributing to discogenic pain (Freemont et al., 2002). Rupture of the weakened AF causes the disc to bulge or prolapse from the vertebral column; impingement of adjacent neural structures causes local pain that often radiates to the extremities. The failure of one disc accelerates the deterioration of adjacent discs and induces pathologic changes in the supporting spinal structures. Conventional therapies (e.g., steroid injection, physical therapy, spinal arthrodesis/fusion), can provide symptomatic relief, but have no effect on disc structure or the underlying degenerative processes.

Studies of disc biology have identified numerous gene products with therapeutic potential in IVDD, but diminished bioactivity in recombinant form and brief half-life *in vivo* limit their efficacy. Moreover, the insertion of a needle into the IVD can initiate or exacerbate disc degeneration and is used routinely to induce experimental IVDD (Carragee et al., 2009; Cuellar et al., 2016). While adverse effects can be minimized with careful technique (Kang, 2010), approaches requiring repeat injection are ill-suited to IVDD. Gene transfer technologies, however, provide the capacity for sustained targeted delivery of therapeutic gene products with a single injection. The dense ECM has a profound impact on the dispersion of vectors and cells that must be factored into the design of the treatment strategy.

Investigations of IVDD gene therapy follow two directions: 1) blocking degenerative progression by inhibiting inflammatory signaling and/or ECM proteolysis, or 2) repair/regeneration by increasing the cellularity and ECM synthesis. The best delivery strategies remain unclear. Direct intra-discal injection of recombinant vectors provides ease of application, while *ex vivo* approaches, in addition to transgenic expression provide supplemental cells to augment the biosynthetic capacity of degenerate disc.

Initial studies of IVD gene transfer involved intra-discal injection of a first-generation Ad vector containing the LacZ reporter gene (Nishida et al., 1998). Histologic assays showed effective transduction of NP cells, but reporter activity was limited to cell populations adjacent to the needle track. Remarkably, despite the use of an immunogenic vector and transgene, reporter activity was sustained for 12 months, demonstrating the quiescence of the NP cells *in situ* and their sequestration from immune surveillance (Nishida et al., 1998). Despite limited vector dissemination, enhanced matrix production and protection has been reported in experimental models of IVDD following intra-discal gene delivery of factors, such as TGF-β1 (Nishida et al., 1999), TGF-β3, IGF-1, BMP-2 (Leckie et al., 2012), GDF-5 (Liang et al., 2010), and TIMP-1 (Leckie et al., 2012; Yue et al., 2016) and various RNAi molecules with several viral vectors, including recombinant AAV, lentivirus and adenovirus. If the IVD ECM provides a barrier to lymphocyte populations then it will similarly obstruct the entry of and dispersion of injected MSCs.

Existing data indicate that AAV and Ad vectors, and by extension lentiviral vectors, are unable to penetrate and traverse the NP matrix (Nishida et al., 1998; Nishida et al., 1999; Lattermann et al., 2005). Dense concentrations of proteoglycans and glycosaminoglycan chains impede the entry of viral particles and form an impenetrable barrier to exogenous cells. Due to the stiffness and low permeability of the ECM, the injection of a fluid volume can increase the local pressures and force the expulsion of suspensions of vector or cells backward through the needle track (Varden et al., 2019) to engage off-target tissues. Studies of ectopic growth factor expression following vector leakage from the rabbit disc found that high doses of Ad.TGF-β1 or Ad.BMP-2 induced severe adverse effects on the central nervous system, including lower limb paralysis, loss of sensory perception and chondro-osseous metaplasia (Levicoff et al., 2008; Watson et al., 2010). These toxicities caution against intra-discal gene delivery of anabolic growth factors for IVDD (Kadow et al., 2015).

Regarding *ex vivo* approaches, endogenous subpopulations of notochordal and MSC-like cells are already adapted to the hostile growth environment, but limited supply and invasiveness of harvest likely limit their use in cellular therapies. MSCs from bone marrow or fat are more abundant and accessible and can be induced into an NP-like phenotype *in vitro*. The harsh growth environment and ECM content complicate effective delivery leaving questions of the utility of cell-based therapies in IVDD. Despite these fundamental technical barriers clinical trials are currently investigating the local delivery of unmodified MSCs in IVDD (Crevensten et al., 2004). Tracking studies of GFP-labeled MSCs following intra-discal injection found very few GFP + cells remained within the body of the disc, but instead were predominantly located in osteophytes in the adjacent vertebral bodies, consistent with ectopic colonization following egress from disc (Vadalà et al., 2012). Studies from others show that the limited number of cells retained in the disc are localized in clusters at the injection site (Maidhof et al., 2017) and die within a few weeks of delivery. To date, there is no proven method by which exogenous cells, genetically modified or otherwise, can be delivered to the IVD and enhance the cellular content or substantively contribute to ECM synthesis or repair (Sakai and Andersson, 2015; Tao et al., 2016; Loibl et al., 2019; Binch et al., 2021).

While currently topical, successful use of RNAi or gene editing technologies would require functional modification of a majority of the cells throughout the volume of the IVD. Regardless of the vector type or delivery approach, existing data indicate that intra-discal gene transfer with this level of pervasion and efficiency is currently unachievable (Lin et al., 2021). Due to concerns with leakage post-injection, IVDD does not appear to be a reasonable target for gene delivery of anabolic growth factors, or *ex vivo* methods in general (Kadow et al., 2015). Considering the available data, delivery of cDNAs for secreted anti-inflammatory gene-products without agonist activity, such as IL-1Ra (Le Maitre et al., 2007), currently appear to have the greatest potential for clinical translation.

### Tendon and Ligament

Tendons and ligaments are the prototypical “connective” tissues. Tendons transmit contractile forces of muscle to bones to enable movement and locomotion, while ligaments connect opposing bones at sites of articulation to provide structural stability. As tendon and ligament have related functions, they have similar architectures and are assembled predominately of collagens I and III, which give the tissues tensile strength; proteoglycans, elastic fibers, and water provide viscoelasticity (Zhao et al., 2021). Both ligament and tendon represent hierarchical structures of collagen fiber bundles surrounded by loose connective tissues that provide vasculature and innervation. The resident cell populations (tenocytes and ligament fibroblasts) represent minor components of their respective tissues. Uniformly distributed along the lengths of the collagen fibers they maintain matrix quality and facilitate repair (Docheva et al., 2015). Tendon/ligament injuries and ruptures are common and occur in joints throughout the body, but the anterior cruciate ligament (Grassi et al., 2020), rotator cuff (Tashjian, 2012) and Achilles tendon (Raikin et al., 2013) are among the most commonly affected. The underlying bases for injury arise from a combination of intrinsic influences (e.g., age, muscle weakness, gender, genetics) and extrinsic factors (trauma, nutrition, exercise, smoking, overloading etc.) (Ilaltdinov et al., 2021).

Healthy tendon tissue has the potential to self-repair if the ends of the fractured tissue are in direct contact and the vascular connective tissue remains intact (Docheva et al., 2015). However, the process is extremely slow and inefficient due to the limited vascularity, cellularity, and low growth factor activity. In most patients, especially seniors, ineffective remodeling and maturation (Kader et al., 2002) generate repair tissue resembling poorly aligned scar tissue with reduced tensile strength and increased risk of re- injury (Sharma and Maffulli, 2005).

Throughout the repair process a wide range of growth factors participate in various stages to promote the deposition of different ECM components and stimulate angiogenesis and fibroblast proliferation (Docheva et al., 2015). In efforts to enhance the natural processes, various biological approaches have been examined, including the local delivery of recombinant growth factors, MSCs and biomaterials (cell-seeded or alone), but have failed to enhance the strength or quality of repair tissues.

In the milieu of prolonged repair, gene-based strategies provide the capacity for sustained delivery of factors to stimulate the synthesis of endogenous signaling molecules, transcription factors and ECM components in the target tissue(s). In this respect, most viral vectors have been shown to deliver and express marker genes and reporters with reasonable efficiency to the ligaments and tendons of animal models by both *in vivo* and *ex vivo* methods (Gerich et al., 1997; Hildebrand et al., 1999). Most repair strategies have employed the use of cDNAs for growth factors and proteins associated with tendon/ligament development, including BMP-12/GDF-7, (Lou et al., 2001; Majewski et al., 2008), BMP-14/GDF-5 (Basile et al., 2008; Hasslund et al., 2014), Scleraxis (SCX) (Hsieh et al., 2016), Mowhawk Homeobox (MKX) (Otabe et al., 2015), RUNX-2 (Zhang et al., 2016), periostin (POSTN) (Noack et al., 2014) and tenomodulin (TNMD) (Jiang et al., 2017) alone and in combination. Other cDNAs such as BMP-2, BMP-4 (Coen et al., 2011), Smad-8 (Hoffmann et al., 2006), TGF-β1 (Majewski et al., 2012) VEGF (Tang et al., 2016), and IGF-1 known to increase cellularity, vascularity and ECM deposition have also been tested. The use of gene transfer to aid assimilation of transplanted ligament or tendon tissues to bone following ligament reconstruction surgery has been examined with some success (Martinek et al., 2002; Zhang et al., 2016; Bez et al., 2018).

## Discussion

The proof-of-concept, that gene transfer can be used as a drug delivery system in orthopaedic conditions is well-established in a broad range of disease/injury models. Toward clinical translation, gene therapy for osteoarthritis has progressed furthest, with two phase I studies examining direct intra-articular gene transfer of IL-1Ra. A phase III trial involving local injection of 293 cells modified to express TGF-β1 mixed with allogenic chondrocytes is listed on the clinical trials registry, but its current status is uncertain, and controversy continues to surround this treatment strategy.

Among the other areas, bone repair appears the closest to clinical testing. Many studies have shown that various methods of delivering cDNAs for osteoinductive factors can serve as an autograft substitute or adjunct in critical-sized skeletal defects in rodent models, but remarkably few have progressed into large animals (Southwood et al., 2012). Those that have, primarily addressed issues of feasibility (Ishihara et al., 2010a; Ishihara et al., 2010b) with variable levels of success, indicating that technical issues of scale-up remain to be resolved (Wilkinson et al., 2021). Regarding cartilage repair, little has advanced toward a clinical setting, especially findings relating to genetically modified MSCs.

Among the experimental gene therapies under investigation for skeletal disorders, the wide discrepancies among research groups regarding vectors, transgenes, delivery methods, experimental species, injury and disease models etc. prohibit direct comparisons of efficacy and elucidation of the technologies best-suited to individual applications. The majority of published accounts describe one-off studies involving an arbitrary dose of an uncharacterized vector or inhibitory RNA without supporting pharmacologic data. For the broader field to advance, a marked shift away from phenomenology is desperately needed. For both *in vivo* and *ex vivo* procedures longitudinal cytologic tracking studies are necessary to establish the spatiotemporal patterns of transgene expression among the modified cell populations, and the nature and extent that transgene expression influences the repair environment. In this respect, small animals fail to represent the size and 3-dimensional volume of human tissues and underestimate the challenges of clinical treatment. The use of large animal models is especially important for these types of investigations. Unfortunately, the value and clinical relevance of studies performed in large animals is often unrecognized by grant reviewers as applications receive low scores for not being “hypothesis driven” or sufficiently innovative or mechanistic. These data, though, are pivotal to clinical translation and are required by the FDA for testing advanced therapeutic medicinal products (ATMPs) in human subjects (Ribitsch et al., 2020).
